# Does Digital Literacy Empower Adolescent Girls in Low- and Middle-Income Countries: A Systematic Review

**DOI:** 10.3389/fpubh.2021.761394

**Published:** 2021-12-16

**Authors:** Salima Meherali, Komal Abdul Rahim, Sandra Campbell, Zohra S. Lassi

**Affiliations:** ^1^Faculty of Nursing, University of Alberta, Edmonton Clinic Health Academy, Edmonton, AB, Canada; ^2^Aga Khan University Hospital, Karachi, Pakistan; ^3^John W. Scott Health Sciences Library, University of Alberta, Edmonton, AB, Canada; ^4^Department of Medicine, Robinson Research Institute, The University of Adelaide, Adelaide, SA, Australia

**Keywords:** digital literacy, adolescent, girls, empowerment, low- and middle-income countries (LMICs)

## Abstract

**Background:** The vast majority (90%) of the world's adolescents aged 10–19 live in low- and middle-income countries (LMICs); and in those resource-limited settings, girls face distinct challenges across multiple health, social, and economic domains. Gender equality and girls' empowerment are key goals in their own right and are central to all other development goals. Digital literacy is a great enabler for the empowerment of young girls. This systematic review aims to assess the range and nature of digital literacy interventions implemented to empower adolescent girls in LMICs and identify evidence about adolescent girls' access and use of digital technologies in LMICs.

**Methods:** We conducted a systematic review of studies following Preferred Reporting Items for Systematic Reviews and Meta-analysis (PRISMA) standards for systematic reviews. Two reviewers selected studies, conducted quality assessments, and extracted data by using standard forms. The collected data include the design of the study, type of digital literacy intervention, target audience, intervention received, intervention reach, data analysis, and study outcomes. The review is registered with PROSPERO (CRD42020216756).

**Results:** Thirty-five studies met the eligibility for inclusion and of those, 11 were experimental studies (randomized controlled trial = 6; quasi-experimental = 2; before-after with no control = 3), 11 were cross-sectional/descriptive studies, seven studies used a mixed-method approach, and six were qualitative studies on digital literacy interventions to empower young girls in LMICs. The majority of digital literacy interventions were designed and implemented to improve sexual and reproductive health rights and decision-making of adolescent girls in LMICs (*n* = 33). Only three papers reported the use of digital media for health-related information and decision making, while only one reported on educational and social empowerment.

**Discussion:** Our findings suggest that digital literacy interventions such as mobile phones, mobile health tools, media exposure, access to the internet, internet-based educational strategies, social media exposure are effective to empower adolescent girls to access health services and information and also enhance the access to educational resources. However, we found inconclusive evidence on the effectiveness of digital literacy to enhance girls' access to financial services and economic empowerment. More rigorous studies with long-term follow-ups to assess the effectiveness of such interventions to empower adolescent girls in LMICs are urgently needed.

## Introduction

Digital literacy refers to the individual's ability to use digital platforms and resources to find, evaluate, and obtain information (1). The effects of digital literacy on varied domains of life are inevitable. In the education domain, the knowledge of digital technology can help students become lifelong learners and help to engage them in the process of acquiring academic skills (2). Digital literacy also influences social empowerment; it helps people stay connected and be informed about the things that are revolving around society.

Digital literacy is critical for children's and young people's development across a wide range of areas. These include engagement in online education, both formal and informal learning, access to critical information and support related to health and well-being, and searching for employment, career information, and entrepreneurship opportunities. This holds great importance for young girls and women's empowerment especially in settings where the women lag behind the men and lack the necessities to live (3). In addition, digital literacy fosters creativity, develops critical thinking, improves problem-solving, and facilitates overall development (4). If young girls are equipped with digital literacy it empowers them and enhances confidence and decision-making abilities (7). Digital adoption and use can also offer women, and girls, in particular, opportunities to overcome hurdles they may face in the physical world. Digital access help expand young girls' sense of self, increase civic engagement, raise awareness of their rights, and increase employment opportunities and workforce participation (5–7).

The use of digital technology also promotes accessing and utilizing sexual and reproductive health (SRH) services among young girls. Digital communication opens the gate and offers wider access to SRH information and services such as puberty, menstruation, bodily autonomy, healthy relationships, contraception etc. (8). Adolescent girls and young women like to seek sensitive SRH information through mobile apps, web browsers, and various social media platforms because it can afford privacy and/or anonymity (9). Moreover, telemedicine which is one of the attributes for digital access to services has been shown to overcome the social barriers, behavioral barriers, and geographic barriers in utilizing especially the sexual health services among adolescents while also aiding in self-utilization of these services (10). Varied digital interventions targeting SRH topics amongst various cultures have shown an acceptable mode of SRH intervention delivery and feasibility amongst the adolescent users (11–13).

Globally, ~1.2 billion people are under the age of 10–19 years, 90% of whom live in LMICs (14). Adolescence is a critical period in life, during which people experience extensive biological, psychological, and social changes (15). Creating and sharing knowledge has never been easier than ever before. But the fact remains that many people still lack the skills required to access this information and an inequity gap is growing (16). The benefits of digital literacy can only be realized if people are empowered with the knowledge and skills to access and use them (17). In many LMICs countries, young girls are 25% less likely than men to access digital technology (18). Bringing adolescent girls into the mainstream of the digital revolution can empower them with access, information, choices, and opportunities that they have never had before. Not just for themselves, but their families, communities, and nation (19). For young girls in LMICs, the internet and digital technologies are an open doorway to tangible benefits such as access to health information, education, and employment opportunities (20). Connecting more adolescent girls and helping them gain the benefits of digital literacy will also support them and their families to navigate this global pandemic, and help achieve many of the United Nation's Sustainable Development Goals (21). The purpose of this systematic review was to assess the range and nature of digital literacy interventions implemented to empower adolescent girls in LMICs and identify evidence about adolescent girls' access to and use of digital technologies in LMICs. The further objectives of this review were to identify and evaluate the effectiveness of digital literacy interventions employed to empower adolescent girls in LMICs, understand the approaches and strategies used to delivery of digital literacy interventions, and identify knowledge gaps in those contexts.

## Methodology

This systematic review was conducted following the Preferred Reporting Items for Systematic Reviews and Meta-analysis (PRISMA) guidelines (22), and included the following studies: experimental [randomized (individually or cluster) and non-randomized controlled trials], observational studies with an internal comparison group (cohort—prospective and retrospective and case-control studies), and qualitative studies. The studies that used case reports, case series, editorials, opinion papers, thesis papers, and review papers were excluded. The protocol for this systematic review was registered with PROSPERO—CRD42020216756.

Studies were included on adolescent girls aged 10–19 years living in LMICs defined by the World Bank (23). The studies with broader age groups were also included if they included the sub-group that included girls in the age range of 10–19 years. Studies that described the access to and interventions to increase access to digital technologies, interventions to improve SRH and rights through digital/mobile technology, and interventions to empower adolescent girls i.e., education and economic development through digital technology or digital literacy were included.

The primary outcome of the study included access to digital technologies by adolescent girls in LMICs, and improved decision-making for own health, continuing education, work, marriage, pregnancy, etc. The secondary outcome of the study included improved SRH status i.e., decrease in early marriages, teenage pregnancies, uptake of contraceptive methods, and improvements in overall health status, etc.

The search strategy was made with the research librarian from the University of Alberta (SC). Databases were searched from inception to December 2020. The databases that were used for running the search strategy included. PubMed, Ovid MEDLINE, the Cochrane Central Register of Controlled Trials (CENTRAL), EPOC systematic review database, Cochrane Database of Systematic Reviews, Database of Abstracts of Reviews of Effects, Health Technology Assessment Database, HealthStar, EMBASE (Excerpta Medica), CINAHL (Cumulative Index to Nursing and Allied Health Literature), PsycINFO (Psychological Abstracts), WHO library and other relevant websites (that publish girls empowerment material) and Sociological Abstracts (Supplementary Material). To minimize the chances of missing any relevant papers, the bibliographical list of the included studies and the systematic reviews were hand-searched. The search results were firstly transferred in an Endnote file and were then uploaded on web-based COVIDENCE software following the removal of the duplicates.

COVIDENCE software was used for streamlining the screening process of systematic review from the stage of title and abstract screening and full-text screening (24). Two independent reviewers (KAR and SM) screened all the potential articles. In case of any discrepancy/conflict between the two reviewers, a third reviewer (ZSL) from the team decided for possible inclusion or exclusion of the study at both stages. The papers that passed eligibility by the reviewers were finally included.

Data were extracted from the included studies such as the name of the author, year of publication, study design, the objective of the paper, target population, sample size, intervention type, intervention mode of delivery, outcomes, and key findings (Tables 1, 2; Supplementary Material). One reviewer (KAR) extracted the data from the included studies. A second reviewer (SM/ZSL) verified all the data extracted from the studies and checked for their accuracy and completeness. Any conflicts were resolved through discussion and consensus.

**Table 1:** Study characteristics of interventional studies.

**References**	**Country**	**Study design**	**Target population (n)**	**Intervention delivery strategy**	**Outcomes and key findings**	**Overall quality**
Halpern et al. (32)	China	RCT	15–19 years Total = 1,337 Intervention = 624; Control = 713	Designed Website	The median scores of the overall knowledge and each specific aspect of reproductive health such as reproduction, contraception, condom, sexually transmitted infections (STIs), and human immunodeficiency virus/acquired immune deficiency syndrome (HIV/AIDS) were significantly higher in the intervention group as compared with those in the control group at postintervention (*p* < 0.0001)	High
Castillo-Arcos et al. (34)	Bolivia	RCT	16–24 years Total = 640 Intervention = 321; Control = 319	mHealth (Text messaging)	No evidence use differed between the groups (33% control vs. 37% intervention; adjusted OR 1.19, 95% CI 0.80 to 1.77; *P* = 0.40). There was a borderline significant effect regarding acceptability (63% control vs. 72% intervention; adjusted OR 1.49, 95% CI 0.98 to 2.28; *P* = 0.06).	High
Njuguna et al. (33)	Palestine	RCT	18–24 years Total = 578 Intervention = 289; Control = 289	Text Messages	Intervention group participants were more likely to find at least one method of effective contraception acceptable (31% in the intervention group vs. 17% in the control group, adjusted OR 2.34, 95% CI 1.48–3.68, *p* < 0.001). They had a higher mean knowledge score, were more likely to find the intrauterine device, injection, implant, and patch acceptable	High
Ahmed (35)	Malaysia	RCT	Young adolescents aged 12 years Total = 209 Internet = 101; Conventional = 108	online SRH education: MyCAP (www.myad-olescenthealth.org)	Using the Internet-based method, there was an increase of 3.88 in the mean knowledge score of participants from pre- to post-intervention. The Internet-based method had a greater eta-squared score of 0.59 compared to the conventional method, which had an eta-squared score of 0.41 (*p* < 0.001)	High
Pedrana et al. (36)	Ghana	RCT	Girls 14–24 years of age Total = 498 Intervention = 205; Control = 293	Through mobile-based group	81% of participants engaged with the mHealth program, with no evidence that the program disproportionally reached better-off groups. The program was effective at increasing knowledge of SRH across all strata. Higher levels of engagement were associated with higher knowledge scores up to a year later. There was no significant impact of the program on self-reported pregnancy within subgroups	Moderate
Bajoga et al. (37)	Uganda	RCT	13–19 years old Total = 366 Intervention = 183; Control = 183	Study materials were written and components conducted in English	94% of intervention youth somewhat or strongly agreed that they learned a lot and 93% said they were somewhat or very likely to recommend the program. Although more than two in three youth somewhat or strongly agreed that the program talked too much about sex (70%) and condoms (75%), 89% somewhat or strongly disagreed that “I do not think kids like me should do the CyberSenga program.”	Moderate
Borzekowski et al. (40)	Mexico	CBA	14 and 17 years Total = 193 Intervention = 96; Control = 97	two face-to-face [sessions 1 and 8] and six online [sessions 2 through 7]	Age was associated with pre-to-posttest changes in sexual resilience (β = −6.10, *p* = 0.019), which partially mediated the effect of the intervention on sexual resilience (β = 5.70, *p* = 0.034). Social support was associated with pre-to-posttest changes in risky sexual behavior (β = −0.17, *p* = 0.039).	Moderate
Bishwajit et al. (38)	Kenya and Brazil	q-RCT	School students: Total = 1,272 Kenya: Total = 558 Intervention = 179; Control = 379 Brazil: Total = 714 Intervention = 559; Control = 155	Access to web-based SRH information	More directed feedback tripled the likelihood of correctly reporting the duration of emergency contraception effectiveness. A review of URL logs suggests that the modest results were due to inadequate exposure to educational materials.	Moderate
Ybarra et al. (39)	Kenya	q-RCT	18–24 years Total = 600 Intervention = 300; Control = 300	SMS/ text messages, surveys, and sessions	The use of weekly text messages about HIV prevention and reproductive health significantly increased rates of HIV testing among young Kenyan women and would be feasible to implement widely among school populations. Approximately half of the participants receiving intervention messages were tested within 12 weeks of the intervention, a rate that is almost twice as fast as those participants not receiving intervention messages.	High

*CBA, controlled before-after; CI, confidence intervals; OR, odds ratio; q-RCT, quasi-randomized controlled trial; RCT, randomized controlled trial; SRH, sexual and reproductive health*.

**Table 2:** Study characteristics of observational and non-controlled before after studies.

**Study ID**	**Country**	**Study design**	**Target population (n)**	**Access to digital technologies**	**Outcomes and key findings**	**Overall quality**
Greenleaf et al. (41)	Bangladesh.	Before/after study	Girls 14–19 years Total = 400	Mobile Phone	Postintervention knowledge score (mean 70.8% ± 9.7%) on RH was significantly higher (paired *t* = 69.721, *p* < 0.001) than the pre-intervention knowledge score (mean 44.71% ± 9.13%) with a large effect size (cohen's d = 3.6). The knowledge score on RH was (*p* < 0.001) correlated (+0.636) with SMS response.	Poor
Ibegbulam et al. (42)	Indonesia	Before/after study	16–24 years Total = 235	SMS intervention	The mean knowledge score significantly increased between baseline and follow-up surveys for SRH questions [2.7, (95% CI 2.47, 2.94) vs. 3.4 (95% CI 2.99, 3.81) (*P* = < 0.01)] and smoking-related questions [3.8 (95% CI 3.66, 3.99) vs. 4.1 (95% CI 3.99, 4.28) (*P* = 0.03)]. A majority of participants reported that the SMS intervention increased their knowledge (95%) and were a useful reminder (95%)	High
Mitchell et al. (43)	Nigeria	Cross-sectional survey	15–24 years Total = 5,765	Mass media	71% of the sample was exposed to Family planning messages in the media within the three months preceding the survey. The main sources of media exposure were mobile phones (48%), radio (37%), and television (29%)	Poor
Nwagwu et al. (44)	Bangladesh	Cross-sectional study	15–49 years Total = 9,014 Adolescents 14–19 years Total = 924	Mobile Phone	Women in the slum areas who used a mobile phone for childbirth service seeking, were 4.3 times [OR = 4.250;95% CI = 1.856–9.734] more likely to receive postnatal care for themselves, and those from outside the city-corporation areas were 2.7 times [OR = 2.707;95% CI = 1.712–4.279] more likely to receive postnatal care for the newborn	High
Samosir et al. (46)	Ghana	Cross-sectional study	15–18 years Total = 778	Internet access	Two-thirds (66%) of the in-school youth and approximately half (54%) of the out-of-school youth had previously gone online. Of all these Internet users, 53% had sought online health information, and this percentage did not differ significantly by gender, age, ethnicity, or even school status. Youth reported great interest, high levels of efficacy, and positive perceptions of online health information.	Moderate
Waldman et al. (47)	Burkina Faso	Cross-sectional study	15–49 years Total = 3,215 Adolescents 15–19 years Total = 710	Mobile Phone	7% reported cell phone ownership. Overall, 22% of women reported current modern contraceptive use. Women who owned a cell phone were more likely to report modern contraceptive use than those who did not (29% vs. 15%).	Moderate
Guerrero et al. (48)	Nigeria.	Descriptive study	Females aged 13–18 years Total = 120	Internet access	The adolescent female students use the Internet to seek for information on general health education (*n* = 120, 100%), sexual hygiene (*n* = 71, 59%), abstinence from premarital sex (*n* = 68, 57%), avoidance of sexual abuse (*n* = 67, 56%). Their preference for the Internet includes its privacy (*n* = 115, 96%) and wealth of information (*n* = 111, 92%).	Moderate
Pfeiffer et al. (49)	Uganda	Survey (cross-sectional)	12–18 years Total = 1,503	Mobile Phone	27% currently have cell phones and about half (51%) of all students and 61% of those who owned a cell phone believe that they would access a text messaging-based HIV prevention program if it were available. Other forms of program delivery modality (e.g., Internet, religious organizations, schools) were preferred to text messaging	Moderate
Reynolds et al. (50)	Nigeria	Cross-sectional study (survey)	13–19 years In-school = 1,011 Out-school = 134	Internet access	Girl youth reported using the internet to access SRH information. The internet was a relatively more important source of information for SRH information for out-of-school girls than in-school girls.	High
Wong et al. (51)	Nigeria	Descriptive survey	13–20 years Total = 1,800	Internet access	Parents, textbooks, television, siblings, radio, friends, school teachers, and the internet were the most accessible source of reproductive health information for adolescent girls in Nigeria.	High
Zakar et al. (52)	Indonesia	Cross-sectional data (DHS)	15–49 years Total = 25,929 Adolescents 15–24 years Total = 6,845	ICT	Contraceptive use predominantly occurred in women who were mobile phone owners, access to the internet, and in those who fully participated in household decision-making. The results of this study confirm the significance of the im-pact of information and communication technology and women's empowerment on contraceptive discontinuation.	High
Winskell et al. (53)	Bangladesh	Cross-sectional survey	17–28 years Total = 436	ICT	young girls are interested in health information and information related to SRH and use phones and computers to access information	Moderate
Nwalo et al. (45)	Uganda	Cross-sectional survey	12–18 years Total = 500	Internet access	Over one-third (35% [173]) had used the computer or Internet to find information about HIV/AIDS, and 20% [102] had looked for sexual health information. Among Internet users, searching for HIV/AIDS information on a computer or online was significantly related to using the Internet weekly, emailing, visiting chat rooms, and playing online games. Internet is a promising strategy to deliver low-cost HIV/AIDS risk reduction interventions.	Moderate
Feroz et al. (61)	Nigeria	Qualitative study design	12–30 years Total = 726	Digital media and Mobile Phone	Mobile phone and internet access improve the access to SRH information among adolescents in Nigeria.	High
Badawy et al. (62)	USA and Botswana	Qualitative study	13–18 years Total = 28	Mobile Phone, Social media	Adolescents in all groups discussed peer pressure and connectedness with mobile phones and social media and had a general knowledge of STIs and HIV. The adolescents agreed that adaptation of risk reduction interventions for mobile phone and social media delivery was warranted, and they shared ideas for adaptation by delivering health information	High
Smith et al. (63)	Senegal	Qualitative study	15–25 years Total = 169	Internet and digital media	Findings suggest that Senegalese youth use a heterogeneous mix of media platforms (i.e., television, radio, internet) to access health and SRH information. Digital media help to improve the youth overall health literacy	High
Jack et al. (64)	Malawi	Qualitative study	15–24 years old Total = 108	Mobile Phone	mHealth has the potential to deliver fundamental preventative health messages to adolescents who are difficult to reach, and which cannot be delivered by the current under-resourced and overstretched health facilities.	High
Petersen et al. (65)	Vietnam	Ethnography qualitative study	15–19 years Total = 20	Internet access	Internet is used to assemble sexual information that was not available from other sources such as the family and school. Young people's narratives also show how they use the Internet as a medium for expressing sexual identities and desires	High
Bacchus et al. (8)	Uganda	Qualitative study	16–19 years Total = 12	Global health Web sites	The present study provides many insights into how the young women accessed information about HIV/AIDS through digital technology and how digital technology impacted their investments in the language practices of their classroom, providing an enhanced range of identities for their futures	High
Girl Effect & Women Deliver et al. (54)	Peru	Mixed-method	13–24 years Total = 172 Adolescent 13–17 years Total = 32	Mobile Phone (SMS text messages)	Adolescents prefer to use a mobile phone (SMS) to received SRH information via Development of ARMADILLO (Adolescent/Youth Reproductive Mobile Access and Delivery Initiative for Love and Life Outcomes)	Moderate
Akinfaderin-Agarau et al. (55)	Tanzania	Mixed-method	15 years (60 questionnaire + 8 interviews)	Social Media	Findings show that youth in Dar es Salaam and Mtwara access the internet mainly through mobile phones. Facebook is by far the most popular internet site. Adolescents highlighted their interest in reproductive and sexual health messages and updates being delivered through humorous posts, links, and clips, as well as by youth role models like music stars and actors that are entertaining and reflect up-to-date trends of modern youth culture	Poor
Cornelius et al. (56)	Ecuador	Mixed-method	Females aged 14.8 (±1.8) Total = 188	Digital Technology and Social Media	Nearly every participant (96.6%) expressed interest in a sexual health education program using technology and social media. A majority of participants indicated that they consulted parents (58.3%) regarding sexual health questions. Only a few participants had access to physicians outside of appointments (3.9%), and most desired more sexual health information (87.3%).	Moderate
Glik et al. (57)	Shanghai	Mixed-method	9–17 years and their parents Total = 800	Internet access	low-income-no-Internet children reported significantly lower scores on all dimensions of digital literacy, academic performance, aspirations, perceived efficacy, self-esteem, family, and peer relationships. On the contrary, low-income children with Internet access did not show significant differences from non-low-income groups across all dimensions.	High
Laidlaw et al. (58)	Pakistan	Mixed-method	Total 1,140 women age >25–<35 years 50% of women age >25 years	ICT	The women wanted to receive information on a wide range of issues, from family planning, antenatal care, and childcare to garbage disposal and prevention of domestic violence. Overall, the ICC/ICT was successful in initiating a meaningful “information dialogue” at the community level, where much-needed information was retrieved, negotiated, mediated, and disseminated through intimate and trusted relations.	Poor
Ngo et al. (59)	Kenya	Mixed-method	11–14 years Total = 30 survey, 27 in FGDs, 22 parents in FGDs	Mobile-based games	the game generated considerable interaction and dialogue with parents, siblings, and friends and catalyzed for children to act as advocates for healthful decisions about sex, both within the family and beyond. The game showed a high level of acceptability with parents	High
Norton et al. (60)	Malawi, India, and Rwanda	Mixed-methods	16–24 years Total = 169	Mobile Phone, internet access, Social Media	Adolescent girls and young women use multiple digital platforms to access varied sensitive SRHR information for health care decision making and it also affords privacy and/or anonymity.	High

*HIV/AIDS, human immunodeficiency virus/acquired immunodeficiency syndrome; ICT, information and communication technology; OR, odds ratio; SRH, sexual and reproductive health; STIs, sexually transmitted infections*.

The methodological quality of all the included studies was assessed by using the Mixed Methods Appraisal Tool (MMAT tool). The tool provided ground to assess the methodological quality of five types of study designs including qualitative studies, randomized-controlled trials, non-randomized cohort studies, quantitative descriptive studies, and mixed-method studies (25).

A tailored approach to synthesize the data was employed by utilizing an evidence table (Tables 1, 2). A narrative synthesis of the included studies was carried out. Given the sparse and heterogeneous data such as age group, study setting, intervention, mode of delivery, and outcomes, the findings of this study cannot be meta-analyzed.

## Results

**Figure 1:**
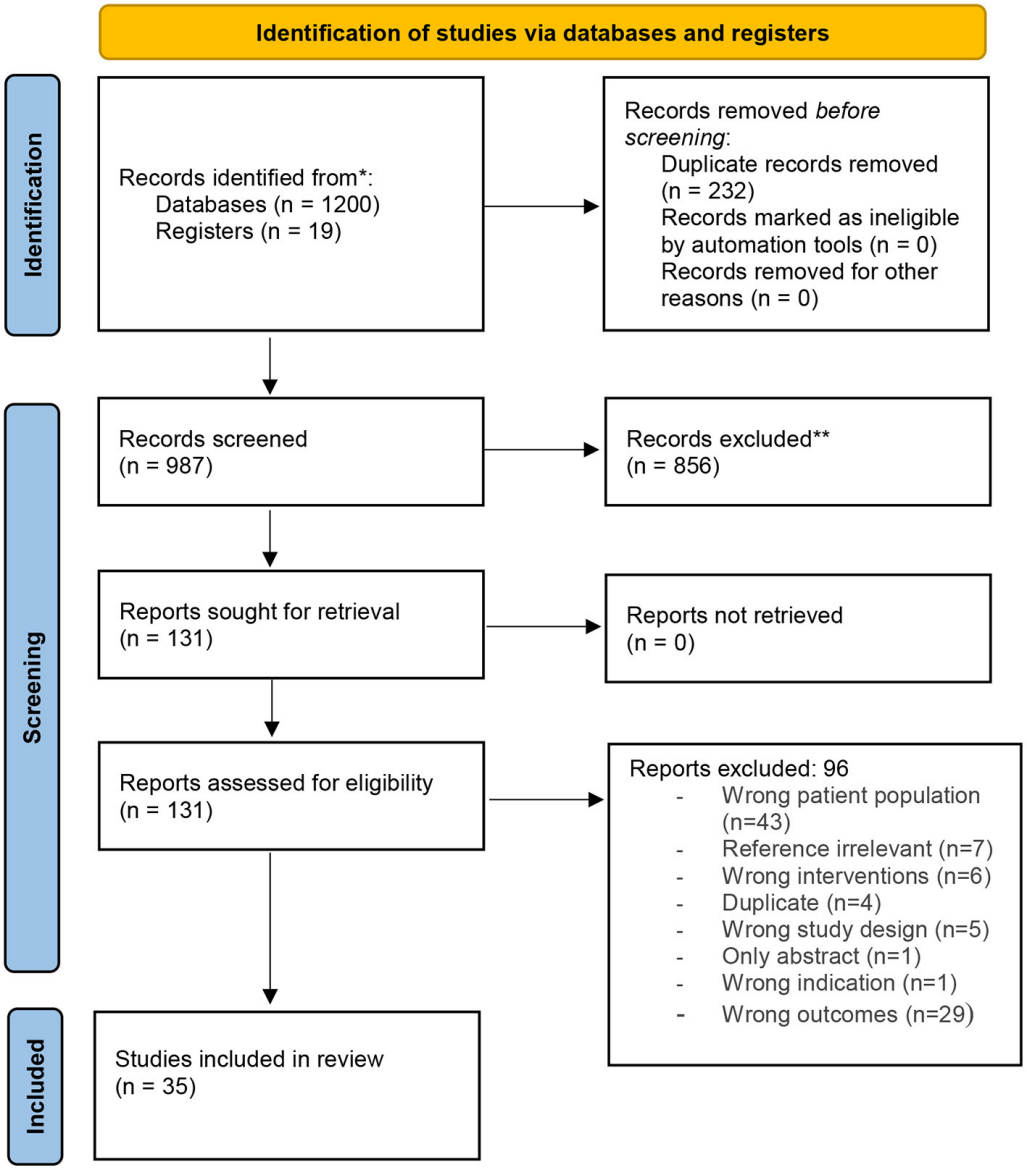
PRISMA for digital literacy.

The initial search retrieved a total of 1,283 articles. After removing duplicates and articles in other languages and reviewing abstracts with respect to the inclusion criteria, a total of 987 studies were considered relevant. Following a full-text review and consultation among the authors, 35 articles were included in the final review and analysis using the PRISMA diagram (Figure 1) (22). Findings from each article were summarized in a table format and systematic analysis was performed to extract major themes. A descriptive synthesis table was formulated containing the textual descriptions of all the findings. A detailed analysis was performed to evaluate the effectiveness of the digital literacy interventions and their impact on adolescent girls' empowerment in LMICs.

A summary of all 35 selected papers, including author, and place of the study, study design, sample size, intervention delivery strategy, and outcomes, are provided in Tables 1, 2. The majority of studies were RCTs (*n* = 6) (26–31), followed by quasi-experimental studies (*n* = 2) (32, 33), before and after studies (*n* = 3) (34–36); while 11 were cross-sectional/descriptive survey design (37–47), seven studies used a mixed-method approach (48–54), and six were qualitative studies (55–60). Sample sizes ranged from 120 to 25,929 subjects in quantitative studies, 12 to 726 participants in qualitative studies, and 68–800 participants in mixed methods studies. Most of the studies have been conducted in the African countries (*n* = 20) (30–33, 37, 39–43, 45, 49, 53–58, 60), 12 studies conducted in Asia (26, 27, 29, 35, 36, 38, 39, 46, 51, 52, 59), three studies conducted in South American countries (34, 38, 54), and one study in North America: Mexico (34). The methodological qualities of included studies are provided in Tables 1, 2. We assessed the methodological quality of the quantitative and qualitative papers using the Mixed-Method Appraisal Tool (M-MAT) (25). The majority of the studies scored moderate to high for interventional studies and poor to high for observational studies. Studies were not excluded based on assessment scores as the purpose was to examine and gain insight into the existing research in this field.

Various digital strategies and interventions were used to empower adolescent girls such as text messages, mHealth, mass media, website development, and mobile-based games. We thematically analyzed and put up the major findings under the thematic areas of digital literacy to empower adolescent girls in LMICs. The studies included were categorized under three headings: digital literacy for sexual and reproductive health and rights empowerment, digital literacy to promote health information and decision making, digital literacy for social and educational empowerment.

### SRH Empowerment

Most of the studies focused on the empowerment of adolescent girls in the area of SRH (*n* = 33) through digital health literacy (26–47, 55, 56). These studies implemented and evaluated the impact of digital literacy interventions on adolescent girls' SRH and STI/HIV knowledge, increase in the usage of contraceptives, and decision making related to contraception, and treatment, and promotion of reproductive health care. Studies that implemented digital literacy interventions to promote SRH knowledge reported that internet-based and mHealth interventions lead to SRH knowledge gain among adolescent girls [Odds Ratio (OR) 6.0; 95% (Confidence Interval (CI) 1.1–32.0)] (26–36), Lou et al. designed a website (www.youthhood.com.cn) that offered SRH knowledge, service information, counseling, and discussion. Participants in the intervention group were only allowed to access the website (26). The study identified that the internet education program increased young girls' (age 15–19 years) SRH health knowledge such as knowledge about reproduction, contraception, condom, STIs, and HIV/AIDS (*p* < 0.0001). The intervention also had a positive influence on their attitudes toward sex-related issues (*p* < 0.05) and toward providing contraceptive service for unmarried people. Similarly, Nik Farid et al. used (MyCAP) website for online SRH education to improve SRH knowledge among Malaysian young people (29). Castillo-Arcos et al. implemented internet-based intervention “Connect” designed to reduce HIV/AIDS sexual risk among Mexican adolescents. Internet-based intervention “Connect” was independently associated with improved self-reported resilience to risky sexual behaviors among Mexican adolescents (34). Halpern et al. implemented the “TeenWeb” project, a multi-year, web-based SRH education intervention implemented in two urban settings: Nairobi, Kenya, and Rio de Janeiro, Brazil. Web-based SRH education intervention increased adolescent girls knowledge about contraception, emergency contraception, HIV testing and prevention, and laws about abortion in their countries (32).

Mobile health or mHealth intervention such as short message services (SMS), and mobile app are also identified as effective tools to increase SRH knowledge of adolescent girls (27, 28, 30, 33, 35, 36, 49, 59), studies reported that mHealth interventions have the potential to engage and increase SRH knowledge of adolescent girls who are at higher risk of poor SRH outcomes, including adolescents with low parental education and support, adolescents with low SRH knowledge, and adolescents with an early sexual debut (27, 28, 30, 33, 35, 36, 58). Njuguna et al. reported that the use of weekly text messages about HIV prevention and reproductive health significantly increased rates of HIV testing among young girls in Kenya (33). McCarthy et al. implemented a mHealth intervention including a mobile app with standard family planning information and intervention messages in two countries i.e., Bolivia and Palestine. The intervention in both countries did not find any difference in the uptake of contraception, whereas, in Palestine, participants reported at least one method of contraceptive acceptable (adjusted OR 2.34; 95% CI 1.48–3.68) (27, 28).

Guerrero et al. conducted participatory research and developed the content for an SMS platform jointly with adolescents and youth in three regions in Peru as part of the ARMADILLO (Adolescent/Youth Reproductive Mobile Access and Delivery Initiative for Love and Life Outcomes) Study. The study reported that adolescents prefer to receive SRH information on various topics such as STI/HIV, SRH rights and policies, pregnancy, and contraceptives through SMS (48).

Most of the studies (*n* = 12) identified that the internet is the main source of information for many adolescents and youth in LMICs. Studies reported that SRH information available on the internet increased the SRH knowledge and improved the attitudes toward SRH of participants (45, 46, 48, 50, 53–55, 58, 60–62, 65). Studies also reported that young girls prefer to use the internet to access SRH information because of its ease of access, low cost, anonymity/privacy, and trustworthiness (39, 40, 42, 44, 45, 47–49, 54–56, 59). Studies reported that information and knowledge adolescent girls gain through digital media empower them to use this information for the prevention of STI/HIV, for decision making on repro-ductive health matters, for self-knowledge, sexual identities, and practices, and the prevention of unplanned pregnancy (39, 44, 45, 47, 52, 54, 60).

Greenleaf et al. examined the association between cell phone ownership and modern contraceptive use in Burkina Faso. The study identified that women with cell phones had 68% higher odds of using a modern contraceptive compared to women with no cell phone. However many adolescent girls (15–19 years) do not own a cell phone in Burkina Faso (41). Bajoga et al. and Samosir et al. also reported that family planning messages via television, radio, and mobile phones are positively associated with safe sexual experience and modern contraceptive methods use among adolescents (37, 46). Cell phone ownership and internet access empower girls and women to use modern contraceptives (43, 46, 54). Mitchell et al. reported that girls who own a cell phone able to access a text messaging-based HIV prevention program and gain more knowledge on HIV prevention (43). Cell phones and social media especially Facebook, Google, WhatsApp, and YouTube are key online resources for adolescent girls and a great source of information on SRH, relationship, pregnancy, contraceptives, STI/HIV prevention, and safer sex practices by adolescent girls (47, 49, 50, 54, 56). Winskell et al. piloted a mobile phone game “*Tumaini”* to prevent HIV among young Africans (11–14 years). The study identified that the game generated interaction and engagement with parents, siblings, and friends and served as a catalyst for children to act as advocates for health decisions about sex, both within the family and beyond (53). Studies also reported that digital literacy improves the utilization of reproductive health services such as family planning, antenatal care, childbirth, postnatal care services, and child care among women of reproductive age (38, 52). In LMICs like Bangladesh, digital literacy helps to reduce maternal and infant mortality among women living in remote and slum areas (38).

### Overall Health Information and Decision-Making Empowerment

Only three studies reported the use of digital literacy for health information and healthcare decision-making (40, 52, 57). Samosir et al. reported that many adolescents in Ghana including girls use the internet to access health information (46). Glik et al. reported that digital literacy and the use of information communication technology are increasing among youth in West Africa. Youth including girls use digital media such as TV, radio, the internet, mobile phone to access health information and use the information for healthcare decision-making. Through digital media, youth keep themselves informed and connected with the outer world (57). Zakar et al. have established an Information and Communication Centre (ICC) in a village in Pakistan where women could access information through online (e.g., internet, mobile phone, etc.) and offline (e.g., CDs, TV, etc.) resources. Women who participated in the project felt satisfied and empowered as otherwise the health care staff rarely took ordinary women's concerns seriously. ICC helped the young women (>25 years) to develop knowledge about, SRH such as information about the availability of contraceptive methods, information about danger signs during pregnancy, etc. women's level of information regarding knowledge about general health issues also increased such as information about the dangers of self-medication, use of disposable syringes for injection, information about causes of hepatitis B and C and regarding garbage disposal. Hence through digital literacy health information reached out to disadvantaged and women with low literacy levels (52).

### Social and Educational Empowerment

We found only one study that explored the impact of digital literacy on adolescents' social and educational empowerment (51). The study examined the difference in the internet access of children with their academic and psychosocial attributes. The study found that children with low-income-no-internet access reported significantly lower scores on all dimensions of digital literacy, various dimensions of development including academic performance, aspirations, perceived efficacy, self-esteem, and family and peer relationships. On the other hand, low-income children with internet access did not show significant differences from non-low-income groups across all dimensions.

## Discussion

This review has identified 35 studies that have assessed the digital literacy interventions and reported outcomes broadly as SRH empowerment, overall health information and decision-making empowerment, and social and educational empowerment.

The findings from this systematic review highlighted that the use of internet and mHealth interventions were found to be effective in improving the knowledge, attitude, and practice toward SRH topics including HIV/STI prevention, and improved uptake of contraception amongst adolescents. Social media especially Facebook, Google, WhatsApp, and YouTube are key online resources for adolescent girls and a great source of information on SRH related topics. Similarly, SMS is a medium for imparting knowledge as well. The odds for accessing the information on SRH topics were significantly higher among girls who owned a mobile phone.

These findings are in concordance with the other reviews, which suggest, that mobile phone approaches; including text messages, in particular, provide feasible and potential effective medium to increase SRH education and ensure safe sexual behaviors among adolescents (61, 62). A Cochrane review on mobile phone-based interventions for improving contraceptive use also suggests that a series of voice messages and daily educational text messages can improve contraceptive use among adolescents (63). Another study also reported that mHealth and digital interventions are not only an effective tool in increasing SRH knowledge, but the digital medium also engages key target populations who are at greater risk of poor SRH outcomes, including adolescents with low parental education, adolescents with low SRH knowledge, adolescents with an early sexual debut, and adolescents with low parental support (30). The internet browsing of sensitive personal information related to SRH or using mobile phones can be very confidential; however, it does vary upon the context (64). There are instances especially in LMICs where mobile phones are being shared within the families and hold the potential to breach the privacy and confidentiality of the SRH accessed information (65). This can also serve as a hindering factor in digital access especially for girls in LMICs lining in resource constraint households (8).

Another important aspect gained from this systematic review is that the use of digital media by adolescent girls not only promotes SRH related outcomes but also improves general well-being and healthcare decision-making. Young girls also access information about the dangers of self-medication, use of disposable syringes for injection, information about causes of hepatitis B and C, and regarding garbage disposal through the digital medium. Similar behavior was seen in school-going adolescent children that they use the internet and other broadband applications for accessing healthcare information. They also used emails to ask for health-related information from the physicians if they had any concerns and also initiate conversations with their teachers pertinent to health-related information (66).

The review also highlighted the effect of digital literacy on social and educational empowerment amongst adolescent girls. These educational attributes were in general but only limited to academic performances, improved self-esteem, and better relationships with significant others. These findings resembled with one of the studies conducted to evaluate the role of digital literacy on performance tests and showed a positive relationship between digital literacy and academic scores. However, after adjusting the confounders, the results were not the same (67). These confounders can be related to classroom-related factors. There is an increased likelihood that the regression analysis of the included study in this systematic review leads to a reverse relationship (non-significant association). Similarly, a study by Girl Effect reports that in contexts where girls have more freedom and agency, they are more likely to have access to digital technology and more opportunities for learning and skill development (68).

While on one hand, digital literacy contributes toward the empowerment of the adolescent population, it also directs them in a negative direction where the youth engage themselves in acts including online harassment, sexual exploitation (69), cyberbullying (70), and pornography (71). Women and girls are at greater risk of digital harm. These digital risks and harms reduce or restrict adolescent girls' digital media use by parents, spouses, and gatekeepers. However, these kinds of issues are less encountered in the nations like LMICs where access to technology is the major hindrance. The absence of mobile, lack of internet connectivity, and the censorship by the parents and government all serve as hindering factors (72). To promote digital/online safety parents and gatekeepers must be informed and educated to help their children on how to stay safe online and what prevention services might be available to them to prevent digital harm and protect users (73).

### Limitations and Future Directions

This systematic review is the first of its kind that has aimed at identifying the effectiveness of digital literacy for empowering girls in LMICs. The findings not only highlighted empowerment in SRH but also gave an insight into the role of digital literacy in promoting general health information and the educational and social aspects. However, there is a need to understand the challenges of data privacy, technological literacy, linguistic competency, and phone access to address the barriers impeding access to digital technology by adolescent girls in LMIC. In addition, a more complete understanding of the role of digital literacy for educational and economic empowerment is required, to strengthen the evidence base in overlooked areas.

While the methodological assessment of the included studies highlighted moderate to the high quality of evidence for interventional studies and poor to the high quality of evidence for observational studies, there were also some limitations. The majority of the included studies were based on cross-sectional/observational study designs. Though these studies help in gauging a snapshot of the exposure and the outcome at a particular time, they do not allow to identify a temporal relationship. Thus, the cross-sectional studies cannot tell the long-term impact or effectiveness of digital literacy on empowering adolescent girls in LMICs. In addition, the interventional studies were not homogenous in terms of intervention used and outcomes reported and therefore pooled estimates and meta-analysis of the could not be performed.

## Conclusion

In the modern era where everything is more digitalized and technology-related, the inequalities based on gender in accessing these are a real concern, especially for girls from disadvantaged settings such as LMICs. The role of digital literacy in empowering adolescent girls living in LMICs is an emerging idea. It not only plays a role in improving the SRH outcomes but also contributes to improving health-related and educational outcomes and decision-making. The findings from this review can be used as a ground for developing new evidence-informed interventions, policies, and practices for girls in LMICs.

## Data Availability Statement

The original contributions presented in the study are included in the article/Supplementary Material, further inquiries can be directed to the corresponding author.

## Author Contributions

SM and ZL participated in the study conception and design. SC searched the articles. SM, ZL, and KR participated in analyses. SM and KR performed the quality assessment and wrote a first draft of the manuscript. ZL commented on this draft and performed critical revisions. All authors have read and approved the manuscript.

## Funding

This project was supported by University of Alberta, Faculty of Nursing VPR SIG Funding 2020.

## Conflict of Interest

The authors declare that the research was conducted in the absence of any commercial or financial relationships that could be construed as a potential conflict of interest.

The reviewer AK declared a shared affiliation with one of the authors, ZL, to the handling editor at the time of review.

## Publisher's Note

All claims expressed in this article are solely those of the authors and do not necessarily represent those of their affiliated organizations, or those of the publisher, the editors and the reviewers. Any product that may be evaluated in this article, or claim that may be made by its manufacturer, is not guaranteed or endorsed by the publisher.
