# Supplementary material for: Does Digital Literacy Empower Adolescent Girls in Low- and Middle-Income Countries: A Systematic Review
Source: Front Public Health. 2021 Dec 16;9:761394. doi: 10.3389/fpubh.2021.761394 (PMC8716589; doi:10.3389/fpubh.2021.761394)
Supplement: Supplementary file 1 [file Table_1.DOCX]

**Supplementary file 1**

**Ovid MEDLINE(R) ALL <1946 to December 30, 2020>**

| **#** | **Search Statement** | **Results** |
| --- | --- | --- |
| 1 | exp *Telemedicine/ or exp *Remote Consultation/ or (telecare or telecollaborat* or teleconsult* or teleconference* or teleeducat* or telehealth or teleguide* or telediagnos* or telelearn* or telemed* or telementor* or telemonitor* or telepresence* or telescreen* or teletransmi* or mhealth or "m heath" or ehealth* or "e health" or mcare or "m care" or tele care or tele collaborat* or tele consult* or tele conference* or tele educat* or tele health or tele guide* or tele diagnos* or tele learn* or tele med* or tele mentor* or tele monitor* or tele presence* or tele screen* or tele transmi* or (sms or phone* or internet or telephone* or texting or "mobile app*" or GMeet* or hangout* or e-mail* or email* or "e chat" or echat or "voice mail*" or "video conferenc*" or "video link*" or "video chat*")).mp. | 270850 |
| 2 | "Mobile Applications"/ or "cell phone"/ or (((smartphone* or cellphone* or "cell phone*" or "smart phone*" or internet or website* or "web page*" or "online influencer*" or "social media" or mobile) adj3 phone*) or mhealth or ehealth or e-health or m-health).mp. | 31623 |
| 3 | (exp Social Media/ or "Text Messaging"/ or (tweets or tweeting or blog or blogging or blogger or texting or "text messag*" or "social media" or "online social network*" or "Baidu Tieba" or Douban or Facebook or Foursquare or "Google app*" or Influenster or Instagram or Kuaishou or Lasso or Linkedin or Messenger or Meetup or Mocospace or Snapchat or "snap chat" or Pinterest or Qzone or "Sina Weibo" or Skype or Steemit or "Tencent QQ" or Tik Tok or Tiktok or Tinder or Tumblr or Twitter or YouTube or "You Tube" or Reddit or Vero or Viber or SMS or VKontakte or Wattpad or Wechat or WhatsApp or Xanga or XING).ti.) not ("SMS 201 995" or "Snyder Robinson Syndrome" or (messenger adj3 RNA)).mp. | 21365 |
| 4 | 1 or 2 or 3 | 285006 |
| 5 | exp Reproductive Rights/ or "Sexual Behavior"/ or (sti or std or "sexually transmitted" or abortion* or "reproductive health" or rape or "forced marriage*" or "child bride*" or "underage marriage*" or contracept* or "birth control" or condom* or pregnan* or menstrua* or (women's adj3 bodies) or (girl's adj3 bodies) or "reproductive safety" or "forced sex*" or prostitut* or "bride price" or dowry or (abduct* adj3 marriag*) or fmg or "genital mutilat*").mp. | 1206401 |
| 6 | ((digital adj3 (empower* or literacy or literate or skill* or comfort)) or ((sexual or sex or reproductive) adj3 (choice* or decision* or deciding or health* or empower* or "self assurance" or "self actualization" or "self actualisation" or "self sureness" or liberation or knowledge or rights or control* or safety or safe or autonomy or independence or information))).ti,ab. | 77629 |
| 7 | 5 or 6 | 1254319 |
| 8 | (girl* or schoolgirl*).mp. [mp=title, abstract, original title, name of substance word, subject heading word, floating sub-heading word, keyword heading word, organism supplementary concept word, protocol supplementary concept word, rare disease supplementary concept word, unique identifier, synonyms] | 151798 |
| 9 | ((youth* or young or adolescen* or pubescen* or marriageable or unmarried) adj2 (woman or women or female* or feminin*)).mp. [mp=title, abstract, original title, name of substance word, subject heading word, floating sub-heading word, keyword heading word, organism supplementary concept word, protocol supplementary concept word, rare disease supplementary concept word, unique identifier, synonyms] | 63644 |
| 10 | female/ or (woman or women or female or feminin*).mp. [mp=title, abstract, original title, name of substance word, subject heading word, floating sub-heading word, keyword heading word, organism supplementary concept word, protocol supplementary concept word, rare disease supplementary concept word, unique identifier, synonyms] | 9157584 |
| 11 | exp Adolescent/ or exp Young Adult/ or exp Child/ | 3445945 |
| 12 | 8 or 9 or (10 and 11) | 2475084 |
| 13 | Developing Countries/ or Africa/ or Africa, Eastern/ or Africa, Southern/ or Africa, Western/ or Africa, Central/ or Africa, Northern/ or "Africa South of the Sahara"/ or Caribbean Region/ or Central America/ or South America/ or Transcaucasia/ or Afghanistan/ or Albania/ or Algeria/ or Angola/ or Antigua/ or Barbuda/ or Argentina/ or Armenia.mp. or Aruba/ or Azerbaijan/ or Bahamas/ or Bahrain/ or Bangladesh/ or Barbados/ or "Republic of Belarus"/ or Belize/ or Benin/ or Bhutan/ or Bolivia/ or "Bosnia and Herzegovina"/ or Botswana/ or Brazil/ or Brunei/ or Bulgaria/ or Burkina Faso/ or Burundi/ or Cambodia/ or Cameroon/ or Cape Verde/ or Central African Republic/ or Chad/ or exp China/ or Chile/ or Colombia/ or Comoros/ or "Democratic People's Republic of Korea"/ or "Democratic Republic of the Congo"/ or Costa Rica/ or Cote d'Ivoire/ or Croatia/ or Cuba/ or Dijbouti/ or Dominica/ or Dominican Republic/ or Ecuador/ or Egypt/ or El Salvador/ or Equatorial Guinea/ or Eritrea/ or Eswatini/ or Ethiopia/ or Fiji/ or French Guiana/ or Gabon/ or Gambia/ or "Georgia (Republic)"/ or Ghana/ or Grenada/ or Guatemala/ or Guinea/ or Guinea Bissau/ or Guyana/ or Haiti/ or Honduras/ or Hungary/ or India/ or Indonesia/ or Iran/ or Iraq/ or Jamaica/ or Jordan/ or Kazakhstan/ or Kenya/ or Kiribati/ or Korea/ or Kuwait/ or Kyrgyzstan/ or Laos/ or Lebanon/ or Lesotho/ or Liberia/ or Libya/ or Madagascar/ or Malawi/ or Malaysia/ or Maldives/ or Mali/ or Marshall Islands/ or Mauritania/ or Mauritius/ or Mexico/ or "Federated States of Micronesia"/ or Moldova/ or Mongolia/ or Montenegro/ or Morocco/ or Mozambique/ or Myanmar/ or Namibia/ or Nauru/ or Nepal/ or Nicaragua/ or Niger/ or Nigeria/ or North Korea/ or "Northern Macedonia"/ or Oman/ or Pakistan/ or Palau/ or Palestine/ or exp Panama/ or Papua New Guinea/ or Paraguay/ or Peru/ or Philippines/ or Qatar/ or Republic of Korea/ or Romania/ or exp Russia/ or Rwanda/ or "Saint Kitts and Nevis"/ or Saint Lucia/ or "Saint Vincent and the Grenadines"/ or Samoa/ or "Sao Tome and Principe"/ or Saudi Arabia/ or Senegal/ or Serbia/ or Seychelles/ or Sierra Leone/ or Solomon Islands/ or Somalia/ or South Africa/ or South Sudan/ or Sri Lanka/ or Sudan/ or Suriname/ or Syria/ or Tajikistan/ or Tanzania/ or Thailand/ or Timor-Leste/ or Togo/ or Tonga/ or "Trinidad and Tobago"/ or Tunisia/ or Turkey/ or Turkmenistan/ or Tuvalu/ or Uganda/ or Ukraine/ or United Arab Emirates/ or Uruguay/ or USSR/ or Uzbekistan/ or Vanuatu/ or Venezuela/ or Vietnam/ or West Indies/ or Yemen/ or Zambia/ or Zimbabwe/ or (Developing Countries or "less developed countries" or "least developed countries" or Caribbean or Central America or South America or Afghanistan or Afghani or Albania or Albanian or Algeria or Algerian or Angola or Angolan or Antigua or Barbuda or Argentina or Armenia or Aruba or Azerbaijan* or Bahamas or Bahamian or Bahrain or Bangladesh or Barbados or Belarus or Belize or Benin or Bhutan or Bhutanese or Bolivia or Bosnia or Herzegovina or Botswana* or Brazil* or Brunei or Bulgaria* or Burkina Faso or Burundi or Cambodia* or Cameroon or Cape Verde or Central African Republic or Chad or China or Chile or Colombia or Comoros or Congo or Costa Rica* or Cote d'Ivoire or Croatia or Cuba or Cuban or Dijbouti or Dominica or Dominican Republic or Ecuador or Egypt or El Salvador or Salvadoran or Equatorial Guinea or Eritrea or Eswatini or Swaziland or Ethiopia or Fiji or French Guiana or Gabon or Gambia or Ghana or Grenada or Guatemala or Guinea or Guinea Bissau or Guyana or Haiti or Haitian or Honduras or Honduran or Hungary or India or Indonesia or Iran or Iranian or Iraq or Iraqui or Jamaica* or Jordan or Kazakhstan* or Kenya or Kenyan or Kiribati or Korea or Korean or Kuwait or Kyrgyzstan* or Laos or Lebanon or Lebanese or Lesotho or Liberia* or Libya or Madagascar or Malawi or Malaysia* or Maldives or Mali or Marshall Islands or Mauritania* or Mauritius or Mexico or Mexican or Micronesia or Moldova or Mongolia* or Montenegr* or Morocco or Mozambique or Myanmar or Namibia* or Nauru or Nepal or Nepalese or Nicaragua or Niger or Nigeria* or Macedonia* or Oman or Pakistan* or Palau or Palestine or Panama or Panamanian or Papua New Guinea or Paraguay or Peru or Peruvian or Philippines or Filipino or Qatar or Romania* or Russia or Rwanda* or "Saint Kitts and Nevis" or Saint Lucia or "Saint Vincent and the Grenadines" or Samoa or "Sao Tome and Principe" or Saudi Arabia* or Senegal* or Serbia* or Seychelles or Sierra Leone or Solomon Islands or Somalia* or South Africa* or Sri Lanka* or Sudan or Sudanese or Suriname or Syria or Syrian or Tajikistan* or Tanzania* or Thailand or Timor-Leste or Togo or Tonga or Trinidad* or Tobago or Tunisia* or Turkey or Turkmenistan* or Tuvalu or Uganda* or Ukraine or United Arab Emirates or Uruguay or Uzbekistan or Vanuatu or Venezuela* or Vietnam* or West Indies or "West Indian" or Yemen* or Zambia* or Zimbabw*).mp. | 1946108 |
| 14 | 4 and 7 and 12 | 4460 |
| 15 | 13 and 14 | 772 |
| 16 | 2 or 3 | 50419 |
| 17 | 7 and 12 and 13 and 16 | 267 |
| 18 | "men who have sex with men".mp. | 11944 |
| 19 | 17 not 18 | 263 |
| 20 | remove duplicates from 19 | 263 |

**Embase <1974 to 2020 December 30>**

| **#** | **Search Statement** | **Results** |
| --- | --- | --- |
| 1 | mobile application/ or mobile health application/ or "cell phone"/ or smartphone/ or (((smartphone* or cellphone* or "cell phone*" or "smart phone*" or internet or website* or "web page*" or "online influencer*" or "social media" or mobile) adj3 phone*) or mhealth or ehealth or e-health or m-health).mp. | 53877 |
| 2 | (social media/ or "text messaging"/ or (tweets or tweeting or blog or blogging or blogger or texting or "text messag*" or "social media" or "online social network*" or "Baidu Tieba" or Douban or Facebook or Foursquare or "Google app*" or Influenster or Instagram or Kuaishou or Lasso or Linkedin or Messenger or Meetup or Mocospace or Snapchat or "snap chat" or Pinterest or Qzone or "Sina Weibo" or Skype or Steemit or "Tencent QQ" or Tik Tok or Tiktok or Tinder or Tumblr or Twitter or YouTube or "You Tube" or Reddit or Vero or Viber or SMS or VKontakte or Wattpad or Wechat or WhatsApp or Xanga or XING).ti.) not ("SMS 201 995" or "Snyder Robinson Syndrome" or (messenger adj3 RNA)).mp. | 36931 |
| 3 | *telemedicine/ or *teleconsultation/ or (telecare or telecollaborat* or teleconsult* or teleconference* or teleeducat* or telehealth or teleguide* or telediagnos* or telelearn* or telemed* or telementor* or telemonitor* or telepresence* or telescreen* or teletransmi* or mhealth or "m heath" or ehealth* or "e health" or mcare or "m care" or tele care or tele collaborat* or tele consult* or tele conference* or tele educat* or tele health or tele guide* or tele diagnos* or tele learn* or tele med* or tele mentor* or tele monitor* or tele presence* or tele screen* or tele transmi* or (sms or phone* or internet or telephone* or texting or "mobile app*" or GMeet* or hangout* or e-mail* or email* or "e chat" or echat or "voice mail*" or "video conferenc*" or "video link*" or "video chat*")).ti. | 69536 |
| 4 | 1 or 2 or 3 | 137373 |
| 5 | Reproductive Rights/ or exp sexual behavior/ or (sti or std or "sexually transmitted" or abortion* or "reproductive health" or rape or "forced marriage*" or "child bride*" or "underage marriage*" or contracept* or "birth control" or condom* or pregnan* or menstrua* or (women's adj3 bodies) or (girl's adj3 bodies) or "reproductive safety" or "forced sex*" or prostitut* or fmg or "genital mutilat*").mp. | 1374244 |
| 6 | ((digital adj3 (empower* or literacy or literate or skill* or comfort)) or ((sexual* or reproductive) adj3 (choice* or decision* or deciding or health* or empower* or "self assurance" or "self actualization" or "self actualisation" or "self sureness" or liberation or knowledge or rights or control* or safety or safe or autonomy or independence or information))).ti,ab. | 58563 |
| 7 | (sex adj2 (choice* or decision* or deciding or health* or empower* or "self assurance" or "self actualization" or "self actualisation" or "self sureness" or liberation or knowledge or rights or control* or safety or safe or autonomy or independence or information)).mp. [mp=title, abstract, heading word, drug trade name, original title, device manufacturer, drug manufacturer, device trade name, keyword, floating subheading word, candidate term word] | 41818 |
| 8 | 5 or 6 or 7 | 1423040 |
| 9 | (girl* or schoolgirl*).mp. [mp=title, abstract, heading word, drug trade name, original title, device manufacturer, drug manufacturer, device trade name, keyword, floating subheading word, candidate term word] | 204783 |
| 10 | ((youth* or young or adolescen* or pubescen* or marriageable or unmarried) adj2 (woman or women or female* or feminin*)).mp. [mp=title, abstract, heading word, drug trade name, original title, device manufacturer, drug manufacturer, device trade name, keyword, floating subheading word, candidate term word] | 83121 |
| 11 | female/ or (woman or women or female or feminin*).mp. [mp=title, abstract, heading word, drug trade name, original title, device manufacturer, drug manufacturer, device trade name, keyword, floating subheading word, candidate term word] | 9855636 |
| 12 | adolescent/ or young adult/ or child/ or juvenile/ | 2865515 |
| 13 | 9 or 10 or (11 and 12) | 1962607 |
| 14 | exp developing country/ or "africa south of the sahara"/ or angola/ or benin/ or burkina faso/ or burundi/ or cameroon/ or cape verde/ or central africa/ or central african republic/ or chad/ or comoros/ or congo/ or cote d'ivoire/ or democratic republic congo/ or djibouti/ or equatorial guinea/ or eritrea/ or ethiopia/ or gabon/ or gambia/ or ghana/ or guinea/ or guinea-bissau/ or kenya/ or lesotho/ or liberia/ or madagascar/ or malawi/ or mali/ or mozambique/ or namibia/ or niger/ or nigeria/ or rwanda/ or senegal/ or sierra leone/ or exp somalia/ or south sudan/ or sudan/ or swaziland/ or tanzania/ or togo/ or uganda/ or zambia/ or zimbabwe/ or South Africa/ or Central Africa/ or north africa/ or algeria/ or egypt/ or libyan arab jamahiriya/ or mauritania/ or morocco/ or tunisia/ or western sahara/ or china/ or far east/ or guangxi/ or hong kong/ or inner mongolia/ or macao/ or ningxia/ or tibet/ or xinjiang/ or korea/ or north korea/ or south korea/ or southeast asia/ or borneo/ or brunei darussalam/ or cambodia/ or laos/ or myanmar/ or papua new guinea/ or thailand/ or timor-leste/ or viet nam/ or irian jaya/ or papua province/ or west papua province/ or Indonesia.mp. or malaysia/ or "federal territory of kuala lumpur"/ or johor/ or kedah/ or kelantan/ or melaka/ or negeri sembilan/ or pahang/ or penang/ or perak/ or perlis/ or sabah/ or sarawak/ or selangor/ or terengganu/ or asia/ or kazakhstan/ or kyrgyzstan/ or tajikistan/ or turkmenistan/ or uzbekistan/ or karakalpakstan/ or middle east/ or bahrain/ or cyprus/ or iran/ or jordan/ or kuwait/ or lebanon/ or oman/ or qatar/ or syrian arab republic/ or "turkey (republic)"/ or yemen/ or iraq/ or iraqi kurdistan/ or united arab emirates/ or abu dhabi/ or ajman/ or dubai/ or sharjah/ or south asia/ or afghanistan/ or bangladesh/ or bhutan/ or nepal/ or sri lanka/ or india/ or "andaman and nicobar islands"/ or andhra pradesh/ or assam/ or bihar/ or chandigarh/ or chhattisgarh/ or goa/ or gujarat/ or haryana/ or himachal pradesh/ or "jammu and kashmir"/ or jharkhand/ or karnataka/ or kerala/ or lakshadweep/ or madhya pradesh/ or maharashtra/ or manipur/ or meghalaya/ or mizoram/ or nagaland/ or "national capital territory of delhi"/ or odisha/ or puducherry/ or "punjab (india)"/ or rajasthan/ or sikkim/ or tamil nadu/ or telangana/ or tripura/ or uttar pradesh/ or uttarakhand/ or west bengal/ or pakistan/ or "azad jammu and kashmir"/ or gilgit-baltistan/ or caribbean islands/ or "anguilla (country)"/ or "antigua and barbuda"/ or aruba/ or bahamas/ or barbados/ or cayman islands/ or cuba/ or curacao/ or dominica/ or dominican republic/ or grenada/ or guadeloupe/ or haiti/ or jamaica/ or martinique/ or montserrat/ or netherlands antilles/ or puerto rico/ or saint barthelemy/ or "saint kitts and nevis"/ or saint lucia/ or "saint vincent and the grenadines"/ or "trinidad and tobago"/ or "turks and caicos islands"/ or "virgin islands (british)"/ or "virgin islands (u.s.)"/ or caribbean netherlands/ or bonaire/ or saba/ or sint eustatius/ or saint martin/ or "saint martin (dutch)"/ or "saint martin (french)"/ or central america/ or belize/ or costa rica/ or el salvador/ or guatemala/ or honduras/ or nicaragua/ or caribbean/ or south america/ or argentina/ or aruba/ or bolivia/ or chile/ or colombia/ or ecuador/ or french guiana/ or guyana/ or paraguay/ or peru/ or suriname/ or uruguay/ or venezuela/ or brazil/ or "acre (state)"/ or alagoas/ or amapa/ or "amazonas (brazil)"/ or bahia/ or ceara/ or espirito santo/ or "federal district (brasilia)"/ or goias/ or maranhao/ or mato grosso/ or mato grosso do sul/ or minas gerais/ or para/ or paraiba/ or "parana (state)"/ or pernambuco/ or piaui/ or "rio de janeiro (state)"/ or rio grande do norte/ or rio grande do sul/ or rondonia/ or roraima/ or "santa catarina (state)"/ or "sao paulo (state)"/ or sergipe/ or tocantins/ or eastern europe/ or albania/ or armenia/ or balkan peninsula/ or belarus/ or bulgaria/ or croatia/ or hungary/ or kosovo/ or moldova/ or "montenegro (republic)"/ or poland/ or "republic of north macedonia"/ or romania/ or slovakia/ or slovenia/ or azerbaijan/ or nagorno karabakh/ or "bosnia and herzegovina"/ or "federation of bosnia and herzegovina"/ or "republic of srpska"/ or "georgia (republic)"/ or abkhazia/ or south ossetia/ or russian federation/ or altai republic/ or bashkortostan/ or buryatia/ or chechnya/ or chukotka/ or chuvashia/ or dagestan/ or evenkia/ or kabardino-balkaria/ or kaliningrad oblast/ or kalmykia/ or khakassia/ or khanty-mansi autonomous [okrug.mp](http://okrug.mp/). or yugra/ or komi republic/ or mari el/ or mordovia/ or nenets autonomous okrug/ or north ossetia-alania/ or "republic of karelia"/ or tatarstan/ or tuva/ or udmurtia/ or yakutia/ or yamalo-nenets autonomus okrug/ or serbia/ or vojvodina/ or ukraine/ or crimea/ or ("developing countr* " or "developing nation " or "developing nations" or "least developed countr*" or Afghanistan or Albania or Algeria or Angola* or Antigua or Argentina or Argentinian or Armenia* or Aruba or Azerbaijan or Bahamas or Bahrain or Bangladesh or Barbados or Barbuda or Basotho or Belarus or Belize or Benin or Bhutan or Bolivia* or Bosnia* or Herzegovina or Botswana* or Brazil or Brunei or Bulgaria or Burkina Faso or Burundi or Cambodia* or Cameroon* or Cape Verde or Cabo Verde or Central African Republic or Chad or Tchad or Chile or Chilean or China or Chinese or Colombia* or Comoros or Congo or Congolese or Costa Rica* or Cote d'Ivoire or Djibouti or Dominica or Dominican Republic or Ecuador* or Egypt* or El Salvador or Salvadoran or Equatorial Guinea or Eritrea* or Eswatini or Ethiopia* or Fiji or Fijian or Gabon or Gambia or (Georgia not (USA or University)) or Ghana or Grenada or Grenadian or Guatemala* or Guinea or Guinea-Bissau or Guyana* or Haiti or Haitian* or Honduras or Honduran or Hungary or India or (Indian not American) or Indonesia* or Iran or Iranian or Iraq or Iraqi or Jamaica* or Jordan or Jordanian or Kazakhstan* or Kenya or Kenyan* or Kiribati or Kosovo or Kosovar or Kuwait* or Kyrgyzstan or Laos or Laotian or Lebanon or Lebanese or Lesotho or Liberia* or Libya or Macedonia* or Madagascar or Malawi or Malaysia* or Maldives or Mali or Marshall Island* or Mauritania or Mauritius or Mexico or Mexican or Micronesia or Moldova or Moldovian or Mongolia* or Montenegro or Montenegran or Morocco or Moroccan or Mozambique or Myanmar or Namibia* or Nauru or Nepal or Nepalese or Nicaragua* or Niger or Nigeria* or Oman or Omani or Pakistan* or Palau or Panama or Panamanian or Papua or New Guinea or Paraguay* or Peru or Peruvian or Philippines or Filipino or Poland or Polish or Qatar or Romania* or Russia* or Rwanda* or Saint Kitts or Nevis or Saint Lucia or Saint Vincent or Grenadines or Samoa* or "SaoTome" or Saudi Arabia* or Senegal* or Serbia* or Seychelles or Sierra or Leone or Solomon Island* or Somalia* or South Africa* or South Sudan* or Sri Lanka* or Sudan or Sudanese or Suriname or Swaziland or Syria* or Tajikistan* or Tanzania* or Thailand or Timor-Leste or Togo or Tonga* or Trinidad* or Tobago or Tunisia or Turkey or Tukish or Turkmenistan* or Tuvalu or Uganda* or Ukraine or "United Arab Emirates" or Uruguay or Uzbekistan or Vanuatu or Venezuela* or Vietnam or Vietnamese or Yemen or Yemeni or Zambia* or Zimbab*).mp. [mp=title, abstract, heading word, drug trade name, original title, device manufacturer, drug manufacturer, device trade name, keyword, floating subheading word, candidate term word] | 2683918 |
| 15 | 4 and 8 and 13 and 14 | 478 |
| 16 | "men who have sex with men".mp. [mp=title, abstract, heading word, drug trade name, original title, device manufacturer, drug manufacturer, device trade name, keyword, floating subheading word, candidate term word] | 18595 |
| 17 | 15 not 16 | 466 |
| 18 | remove duplicates from 17 | 455 |

**Global Health <1910 to 2020 Week 47>**

| **#** | **Search Statement** | **Results** |
| --- | --- | --- |
| 1 | social media/ or "cell phone*".mp. or [cellphone.mp](http://cellphone.mp/). or "smart phone*".mp. or smartphone*.mp. or [mhealth.mp](http://mhealth.mp/). or "m-health".mp. or [ehealth.mp](http://ehealth.mp/). or "e-health".mp. or [telehealth.mp](http://telehealth.mp/). or "tele health".mp. or [internet.mp](http://internet.mp/). or "web page*".mp. or webpage*.mp. or "web site*".mp. or website*.mp. [mp=abstract, title, original title, broad terms, heading words, identifiers, cabicodes] | 23044 |
| 2 | (tweets or tweeting or blog or blogging or blogger or texting or "text messag*" or "social media" or "online social network*" or "Baidu Tieba" or Douban or Facebook or Foursquare or "Google app*" or Influenster or Instagram or Kuaishou or Lasso or Linkedin or Messenger or Meetup or Mocospace or Snapchat or "snap chat" or Pinterest or Qzone or "Sina Weibo" or Skype or Steemit or "Tencent QQ" or Tik Tok or Tiktok or Tinder or Tumblr or Twitter or YouTube or "You Tube" or Reddit or Vero or Viber or SMS or VKontakte or Wattpad or Wechat or WhatsApp or Xanga or XING).ti. not ("SMS 201 995" or "Snyder Robinson Syndrome" or (messenger adj3 RNA)).mp. | 2479 |
| 3 | 1 or 2 | 24349 |
| 4 | ("developing countr* " or "developing nation " or "developing nations" or "least developed countr*" or Afghanistan or Albania or Algeria or Angola* or Antigua or Argentina or Argentinian or Armenia* or Aruba or Azerbaijan or Bahamas or Bahrain or Bangladesh or Barbados or Barbuda or Basotho or Belarus or Belize or Benin or Bhutan or Bolivia* or Bosnia* or Herzegovina or Botswana* or Brazil or Brunei or Bulgaria or Burkina Faso or Burundi or Cambodia* or Cameroon* or Cape Verde or Cabo Verde or Central African Republic or Chad or Tchad or Chile or Chilean or China or Chinese or Colombia* or Comoros or Congo or Congolese or Costa Rica* or Cote d'Ivoire or Djibouti or Dominica or Dominican Republic or Ecuador* or Egypt* or El Salvador or Salvadoran or Equatorial Guinea or Eritrea* or Eswatini or Ethiopia* or Fiji or Fijian or Gabon or Gambia or (Georgia not (USA or University)) or Ghana or Grenada or Grenadian or Guatemala* or Guinea or Guinea-Bissau or Guyana* or Haiti or Haitian* or Honduras or Honduran or Hungary or India or (Indian not American) or Indonesia* or Iran or Iranian or Iraq or Iraqi or Jamaica* or Jordan or Jordanian or Kazakhstan* or Kenya or Kenyan* or Kiribati or Kosovo or Kosovar or Kuwait* or Kyrgyzstan or Laos or Laotian or Lebanon or Lebanese or Lesotho or Liberia* or Libya or Macedonia* or Madagascar or Malawi or Malaysia* or Maldives or Mali or Marshall Island* or Mauritania or Mauritius or Mexico or Mexican or Micronesia or Moldova or Moldovian or Mongolia* or Montenegro or Montenegran or Morocco or Moroccan or Mozambique or Myanmar or Namibia* or Nauru or Nepal or Nepalese or Nicaragua* or Niger or Nigeria* or Oman or Omani or Pakistan* or Palau or Panama or Panamanian or Papua or New Guinea or Paraguay* or Peru or Peruvian or Philippines or Filipino or Poland or Polish or Qatar or Romania* or Russia* or Rwanda* or Saint Kitts or Nevis or Saint Lucia or Saint Vincent or Grenadines or Samoa* or "SaoTome" or Saudi Arabia* or Senegal* or Serbia* or Seychelles or Sierra or Leone or Solomon Island* or Somalia* or South Africa* or South Sudan* or Sri Lanka* or Sudan or Sudanese or Suriname or Swaziland or Syria* or Tajikistan* or Tanzania* or Thailand or Timor-Leste or Togo or Tonga* or Trinidad* or Tobago or Tunisia or Turkey or Tukish or Turkmenistan* or Tuvalu or Uganda* or Ukraine or "United Arab Emirates" or Uruguay or Uzbekistan or Vanuatu or Venezuela* or Vietnam or Vietnamese or Yemen or Yemeni or Zambia* or Zimbab*).mp. | 1262683 |
| 5 | developing countries/ or algeria/ or angola/ or anguilla island/ or "antigua and barbuda"/ or argentina/ or aruba/ or bahamas/ or bahrain/ or barbados/ or belize/ or bermuda/ or bolivia/ or bonaire/ or brazil/ or british virgin islands/ or brunei darussalam/ or cameroon/ or cayman islands/ or chile/ or china/ or "christmas island (indian ocean)"/ or cocos islands/ or colombia/ or congo/ or cook islands/ or costa rica/ or cote d'ivoire/ or crozet islands/ or cuba/ or curacao/ or cyprus/ or dominica/ or dominican republic/ or easter island/ or ecuador/ or egypt/ or el salvador/ or falkland islands/ or "federated states of micronesia"/ or fiji/ or french guiana/ or gabon/ or gambier islands/ or ghana/ or grenada/ or guadeloupe/ or guam/ or guatemala/ or guyana/ or honduras/ or india/ or indonesia/ or iran/ or iraq/ or jamaica/ or jordan/ or kenya/ or kerguelen archipelago/ or korea democratic people's republic/ or korea republic/ or least developed countries/ or lebanon/ or libya/ or malaysia/ or marquesas islands/ or marshall islands/ or martinique/ or mauritius/ or mayotte/ or mexico/ or midway islands/ or mongolia/ or montserrat/ or morocco/ or namibia/ or new britain/ or new caledonia/ or new ireland/ or nicaragua/ or nigeria/ or niue/ or northern mariana islands/ or oman/ or pakistan/ or palau/ or panama/ or papua new guinea/ or paraguay/ or peru/ or philippines/ or puerto rico/ or qatar/ or reunion/ or saba/ or saint helena/ or "saint kitts and nevis"/ or saint lucia/ or "saint vincent and the grenadines"/ or senegal/ or seychelles/ or sri lanka/ or suriname/ or swaziland/ or syria/ or tahiti/ or thailand/ or tokelau/ or tonga/ or "trinidad and tobago"/ or tuamotu/ or tubuai islands/ or tunisia/ or turkey/ or "turks and caicos islands"/ or uruguay/ or venezuela/ or vietnam/ or "wallis and futuna"/ or western sahara/ | 1026715 |
| 6 | 4 or 5 | 1262683 |
| 7 | 3 and 6 | 6739 |
| 8 | (Youth or youths or teen* or pre-teen* or adolesc* or pubescen* or pre-pubescen* or tween* or "high school*" or "middle school*" or marriagable or "young adult*").mp. [mp=abstract, title, original title, broad terms, heading words, identifiers, cabicodes] | 127119 |
| 9 | females/ or (female* or feminin* or pregnan* or menstru*).mp. [mp=abstract, title, original title, broad terms, heading words, identifiers, cabicodes] | 372879 |
| 10 | girls/ or prepubertal females/ or (girl* or schoolgirl*).mp. | 52724 |
| 11 | 8 and 9 | 29320 |
| 12 | 10 or 11 | 74874 |
| 13 | Sexual health/ or reproductive health/ or ((sex or sexual* or reproductive) adj3 (empower* or liberation or safe or safety or control or choice* or decision* or deciding or health or education or autonomy or independence or "self actuali*" or "self sureness")).mp. [mp=abstract, title, original title, broad terms, heading words, identifiers, cabicodes] | 26602 |
| 14 | (digital* adj3 (empower* or education or information or literate or literacy)).mp. [mp=abstract, title, original title, broad terms, heading words, identifiers, cabicodes] | 186 |
| 15 | ("child bride*" or pregnan* or "underage marriage*" or prostitut* or sti or std or "sexually transmitted" or condom* or "birth control" or contracept* or abortion or "bride price" or "forced marriage" or dowry or "forced sex" or (abduct* adj3 marriage*) or rape or honor killing* or honour killing* or "bride burning" or domestic violence or "intimate partner violence" or IPV).mp. | 199104 |
| 16 | 13 or 14 or 15 | 211145 |
| 17 | 7 and 12 and 16 | 79 |

**EBSCO CINAHL with full text Searched December 30, 2020**

Expanders - Apply equivalent subjects

Search modes - Find all my search term

| **#** | **Query** | **Results** |
| --- | --- | --- |
| S1 | ("cell phone*" or cellphone* or smartphone* or "smart phone*" or internet or webpage* or "web page*" or website* or "web site*" or mhealth or ehealth or telehealth or "tele health" or "m health" or "e health" or app or apps or tweets or tweeting or blog or blogging or blogger or texting or "text messag*" or "social media" or "online social network*" or "Baidu Tieba" or Douban or Facebook or Foursquare or "Google app*" or Influenster or Instagram or Kuaishou or Lasso or Linkedin or "Messenger" or Meetup or Mocospace or Snapchat or "snap chat" or Pinterest or Qzone or "Sina Weibo" or Skype or Steemit or "Tencent QQ" or Tik Tok or Tiktok or Tinder or Tumblr or Twitter or YouTube or "You Tube" or Reddit or Vero or Viber or SMS or VKontakte or Wattpad or Wechat or WhatsApp or Xanga or XING) | 41,450 |
| S2 | (MH "Web Search Engines") OR (MH "World Wide Web Applications+") OR (MH "World Wide Web+") OR (MH "Web Browsers") OR (MH "Twitter") OR (MH "Facebook") OR (MH "Social Media+") OR (MH "Internet Access") OR (MH "Internet+") OR (MH "Email") | 1,872 |
| S3 | S1 or S2 | 53,176 |
| S4 | (MH "Developing Countries") OR (MH "Africa+") OR (MH "Africa, Northern+") OR (MH "Algeria") OR (MH "Egypt") OR (MH "Libya") OR (MH "Morocco") OR (MH "Tunisia") OR (MH "Africa South of the Sahara+") OR (MH "Africa, Central+") OR (MH "Cameroon") OR (MH "Central African Republic") OR (MH "Chad") OR (MH "Congo") OR (MH "Equatorial Guinea") OR (MH "Gabon") OR (MH "Africa, Eastern+") OR (MH "Burundi") OR (MH "Djibouti") OR (MH "Eritrea") OR (MH "Ethiopia") OR (MH "Kenya") OR (MH "Rwanda") OR (MH "Somalia") OR (MH "Sudan") OR (MH "Tanzania") OR (MH "Uganda") OR (MH "Africa, Southern+") OR (MH "Botswana") OR (MH "Lesotho") OR (MH "Malawi") OR (MH "Mozambique") OR (MH "Namibia") OR (MH "South Africa") OR (MH "Swaziland") OR (MH "Zambia") OR (MH "Zimbabwe") OR (MH "Africa, Western+") OR (MH "Benin") OR (MH "Burkina Faso") OR (MH "Cape Verde") OR (MH "Cote d'Ivoire") OR (MH "Gambia") OR (MH "Ghana") OR (MH "Guinea") OR (MH "Guinea-Bissau") OR (MH "Liberia") OR (MH "Mali") OR (MH "Mauritania") OR (MH "Niger") OR (MH "Nigeria") OR (MH "Senegal") OR (MH "Sierra Leone") OR (MH "Togo") or (MH "West Indies+") OR (MH "Central America+") OR (MH "Belize") OR (MH "Costa Rica") OR (MH "El Salvador") OR (MH "Guatemala") OR (MH "Honduras") OR (MH "Nicaragua") OR (MH "Panama+") OR (MH "Latin America") OR (MH "South America+") OR (MH "Argentina") OR (MH "Bolivia") OR (MH "Brazil") OR (MH "Chile") OR (MH "Colombia") OR (MH "Ecuador") OR (MH "French Guiana") OR (MH "Guyana") OR (MH "Paraguay") OR (MH "Peru") OR (MH "Suriname") OR (MH "Uruguay") OR (MH "Venezuela") OR (MH "Antigua") OR (MH "Bahamas") OR (MH "Barbados") OR (MH "Cuba") OR (MH "Dominica") OR (MH "Dominican Republic") OR (MH "Haiti") OR (MH "Jamaica") OR (MH "Martinique") OR (MH "Netherlands Antilles") OR (MH "Puerto Rico") OR (MH "Trinidad and Tobago") OR (MH "Virgin Islands of the United States") or (MH "Asia, Central+") OR (MH "Kazakhstan") OR (MH "Kyrgyzstan") OR (MH "Tajikistan") OR (MH "Turkmenistan") OR (MH "Uzbekistan") OR (MH "Asia, Southeastern+") OR (MH "Borneo") OR (MH "Brunei") OR (MH "East Timor") OR (MH "Indonesia") OR (MH "Laos") OR (MH "Malaysia") OR (MH "Myanmar") OR (MH "Philippines") OR (MH "Singapore") OR (MH "Thailand") OR (MH "Timor") OR (MH "Vietnam") OR (MH "Asia, Western+") OR (MH "Bangladesh") OR (MH "Bhutan") OR (MH "India") OR (MH "Middle East+") OR (MH "Afghanistan") OR (MH "Bahrain") OR (MH "Iran") OR (MH "Iraq") OR (MH "Lebanon") OR (MH "Oman") OR (MH "Qatar") OR (MH "Syria") OR (MH "Turkey") OR (MH "Yemen") OR (MH "Nepal") OR (MH "Pakistan") OR (MH "Sri Lanka") OR (MH "Far East+") OR (MH "China+") OR (MH "Hong Kong") OR (MH "Macao") OR (MH "Mongolia") OR (MH "North Korea") OR (MH "South Korea") OR (MH "Taiwan") OR (MH "Pacific Islands+") OR (MH "Melanesia+") OR (MH "Micronesia+") OR (MH "Polynesia+") or "developing countr*" or "developing nation" or "developing nations" or Afghanistan or Albania or Algeria or Angola or Antigua or Barbuda or Argentina or Armenia or Aruba or Azerbaijan or Bahamas or Bahrain or Bangladesh or Barbados or Belarus or Belize or Benin or Bhutan or Bolivia or Bosnia or Herzegovina or Botswana or Brazil or Brunei or Bulgaria or Burkina Faso or Burundi or Cambodia or Cameroon or “Cape Verde” or “Central African Republic” or Chad or Chile or China or Colombia or Comoros or Democratic or Congo or “Costa Rica” or “Cote d'Ivoire” or Djibouti or Dominica or Dominican Republic or Ecuador or Egypt or “El Salvador” or “Equatorial Guinea” or Eritrea or Eswatini or Ethiopia or Fiji or Gabon or Gambia or (Georgia not (USA or University)) or Ghana or Grenada or Guatemala or Guinea or Guinea-Bissau or Guyana or Haiti or Honduras or Hungary or India or Indonesia or Iran or Iraq or Jamaica or Jordan or Kazakhstan or Kenya or Kiribati or Kosovo or Kuwait or Kyrgyzstan or Laos or Lebanon or Lesotho or Liberia or Libya or Macedonia or Madagascar or Malawi or Malaysia or Maldives or Mali or "Marshall Islands" or Mauritania or Mauritius or Mexico or Micronesia or Moldova or Mongolia or Montenegro or Morocco or Mozambique or Myanmar or Namibia or Nauru or Nepal or Nicaragua or Niger or Nigeria or Oman or Pakistan or Palau or Panama or Papua or “New Guinea” or Paraguay or Peru or Philippines or Poland or Qatar or Romania or Russia or Rwanda or "Saint Kitts" or Nevis or "Saint Lucia" or "Saint Vincent" or Grenadines or Samoa or "Sao Tome" or "Saudi Arabia*" or Senegal* or Serbia or Seychelles or "Sierra Leone" or "Solomon Islands" or Somalia or "South Africa" or “South Sudan” or “Sri Lanka” or Sudan or Suriname or Swaziland or Syria or Tajikistan or Tanzania or Thailand or Timor-Leste or Togo or Tonga or Trinidad or Tobago or Tunisia or Turkey or Turkmenistan or Tuvalu or Uganda or Ukraine or United Arab Emirates or Uruguay or Uzbekistan or Vanuatu or Venezuela or Vietnam or Yemen or Zambia or Zimbabwe | Display |
| S5 | (MH "Reproductive Health") OR (MH "Sexual Health") OR (MH "Women's Health") or (MH "Circumcision, Female") | 52,546 |
| S6 | "underage marriage*" or "forced marriage*" or "child bride*" or "honor killing*" or "honour killing*" or (abduct* N3 marriage*) or "forced sex*" or prostitut* or "bride burning" or "domestic violence" or "intimate partner violence" or IPV or dowry or fmg or "genital mutilation*" or sti or "sexually transmitted" or std or condom* or contracept* or "birth control*" | 79,603 |
| S7 | (sex) N2 (empower* or control* or choice* or decision* or deciding or decide or autonomy or independence or rights or "self assurance" or liberation or "self actuali*" or "self sureness" or education or health) or (sexual* or reproductive) N3 (empower* or control* or choice* or decision* or deciding or decide or autonomy or independence or rights or "self assurance" or liberation or "self actuali*" or "self sureness" or education or health) | 45,089 |
| S8 | digital* n2 (empower* or literate or literacy or information or education) | 1,203 |
| S9 | S5 OR S6 OR S7 OR S8 | 148,473 |
| S10 | (MH "Child Abuse, Sexual") OR (MH "Adolescence+") OR (MH "Child+") OR (MH "Latchkey Children") OR (MH "Minors (Legal)") OR (MH "Young Adult") | 1,104,203 |
| S11 | child* or youth or youths or adolescen* or pubescen* or prepubescen* or "high school*" or "middle school* or teen* or tween or tweenie* or tweens or "young adult*" | 1,138,882 |
| S12 | S10 OR S11 | 1,317,312 |
| S13 | girl* or schoolgirl* or "young women" or "young woman" or marriageable or female* or feminin* or menstru* or pregnan* | 2,083,673 |
| S14 | [MH] Female | 787 |
| S15 | S13 OR S14 | 2,083,673 |
| S16 | S3 AND S4 AND S9 AND S12 AND S15 | 320 |
| S17 | "men who have sex with men" | 6,581 |
| S18 | s16 NOT s17 | 312 |

**SOCINDEX Searched December 30, 2020**

Expanders - apply equivalent subject headings

Search Modes - find all my search terms

| \| **#** \| **Query** \| **Results** \| \| --- \| --- \| --- \| \| S1 \| ("cell phone*" or cellphone* or smartphone* or "smart phone*" or internet or webpage* or "web page*" or website* or "web site*" or mhealth or ehealth or telehealth or "tele health" or "m health" or "e health" or app or apps or tweets or tweeting or blog or blogging or blogger or texting or "text messag*" or "social media" or "online social network*" or "Baidu Tieba" or Douban or Facebook or Foursquare or "Google app*" or Influenster or Instagram or Kuaishou or Lasso or Linkedin or "Messenger" or Meetup or Mocospace or Snapchat or "snap chat" or Pinterest or Qzone or "Sina Weibo" or Skype or Steemit or "Tencent QQ" or Tik Tok or Tiktok or Tinder or Tumblr or Twitter or YouTube or "You Tube" or Reddit or Vero or Viber or SMS or VKontakte or Wattpad or Wechat or WhatsApp or Xanga or XING) \| 41,459 \| \| S2 \| DE ("Internet" or "social media" or "social media in medicine" or "cell phones" or "Twitter (Web resource)") OR (SU "Facebook") OR (DE "Social Media+") OR DE "INTERNET" OR DE "CYBERCULTURE" OR DE "EMAIL" OR DE "ONLINE chat" OR DE "WORLD Wide Web" \| 13,457 \| \| S3 \| S1 or S2 \| 53,212 \| \| S4 \| "developing nations" or Afghanistan or Albania or Algeria or Angola or Antigua or Barbuda or Argentina or Armenia or Aruba or Azerbaijan or Bahamas or Bahrain or Bangladesh or Barbados or Belarus or Belize or Benin or Bhutan or Bolivia or Bosnia or Herzegovina or Botswana or Brazil or Brunei or Bulgaria or "Burkina Faso" or Burundi or Cambodia or Cameroon or “Cape Verde” or “Central African Republic” or Chad or Chile or China or Colombia or Comoros or Democratic or Congo or “Costa Rica” or “Cote d'Ivoire” or Djibouti or Dominica or "Dominican Republic" or Ecuador or Egypt or “El Salvador” or “Equatorial Guinea” or Eritrea or Eswatini or Ethiopia or Fiji or Gabon or Gambia or (Georgia not (USA or University)) or Ghana or Grenada or Guatemala or Guinea or Guinea-Bissau or Guyana or Haiti or Honduras or Hungary or India or Indonesia or Iran or Iraq or Jamaica or Jordan or Kazakhstan or Kenya or Kiribati or Kosovo or Kuwait or Kyrgyzstan or Laos or Lebanon or Lesotho or Liberia or Libya or Macedonia or Madagascar or Malawi or Malaysia or Maldives or Mali or "Marshall Islands" or Mauritania or Mauritius or Mexico or Micronesia or Moldova or Mongolia or Montenegro or Morocco or Mozambique or Myanmar or Namibia or Nauru or Nepal or Nicaragua or Niger or Nigeria or Oman or Pakistan or Palau or Panama or Papua or “New Guinea” or Paraguay or Peru or Philippines or Poland or Qatar or Romania or Russia or Rwanda or "Saint Kitts" or Nevis or "Saint Lucia" or "Saint Vincent" or Grenadines or Samoa or "Sao Tome" or "Saudi Arabia*" or Senegal* or Serbia or Seychelles or "Sierra Leone"or "Solomon Islands" or Somalia or "South Africa" or “South Sudan” or “Sri Lanka” or Sudan or Suriname or Swaziland or Syria or Tajikistan or Tanzania or Thailand or Timor-Leste or Togo or Tonga or Trinidad or Tobago or Tunisia or Turkey or Turkmenistan or Tuvalu or Uganda or Ukraine or United Arab Emirates or Uruguay or Uzbekistan or Vanuatu or Venezuela or Vietnam or Yemen or Zambia or Zimbabwe \| 413,734 \| \| S5 \| DE "SEXUAL health" OR DE "CONDOMS" OR DE "PROMISCUITY" OR DE "SAFE sex in AIDS prevention" OR DE "CONDOMS" OR DE "PROMISCUITY" OR DE "REPRODUCTIVE health" OR DE "SAFE sex" OR DE "SEX education" OR DE "SEXUALLY transmitted diseases" or DE "FEMALE genital mutilation" OR DE "INFIBULATION" DE "SEXUAL rights" OR DE "ABORTION" OR DE "BIRTH control" OR DE "CONTRACEPTION" \| 17,831 \| \| S6 \| "underage marriage*" or "forced marriage*" or "child bride*" or "honor killing*" or "honour killing*" or (abduct* N3 marriage*) or "forced sex*" or prostitut* or "bride burning" or "domestic violence" or "intimate partner violence" or IPV or dowry or fmg or "genital mutilation*" or sti or "sexually transmitted" or std or condom* or contracept* or "birth control*" \| 49,394 \| \| S7 \| (sex) N2 (empower* or control* or choice* or decision* or deciding or decide or autonomy or independence or rights or "self assurance" or liberation or "self actuali*" or "self sureness" or education or health) or (sexual* or reproductive) N3 (empower* or control* or choice* or decision* or deciding or decide or autonomy or independence or rights or "self assurance" or liberation or "self actuali*" or "self sureness" or education or health) \| 21,999 \| \| S8 \| digital* n2 (empower* or literate or literacy) \| 278 \| \| S9 \| S5 OR S6 OR S7 OR S8 \| 69,984 \| \| S10 \| DE "CHILDREN" OR DE "ABUSED children" OR DE "ADOPTED children" OR DE "ADULT children" OR DE "ADULT children of alcoholics" OR DE "ADVERTISING & children" OR DE "AIDS & children" OR DE "ARTS & children" OR DE "BAHAI children" OR DE "BILINGUALISM in children" OR DE "BIRTH order" OR DE "BLACK children" OR DE "BOYS" OR DE "BUDDHIST children" OR DE "CHILD beauty pageant contestants" OR DE "CHILD development" OR DE "CHILD patients" OR DE "CHILD prostitutes" OR DE "CHILDREN & erotica" OR DE "CHILDREN & genocide" OR DE "CHILDREN & the environment" OR DE "CHILDREN & war" OR DE "CHILDREN of AIDS patients" OR DE "CHILDREN of Holocaust survivors" OR DE "CHILDREN of LGBTQ+ parents" OR DE "CHILDREN of abused wives" OR DE "CHILDREN of attention-deficit-disordered parents" OR DE "CHILDREN of divorced parents" OR DE "CHILDREN of heterosexual parents" OR DE "CHILDREN of immigrants" OR DE "CHILDREN of intercountry marriage" OR DE "CHILDREN of interethnic marriage" OR DE "CHILDREN of interfaith marriage" OR DE "CHILDREN of migrant laborers" OR DE "CHILDREN of minorities" OR DE "CHILDREN of older parents" OR DE "CHILDREN of parents with disabilities" OR DE "CHILDREN of prostitutes" OR DE "CHILDREN of single parents" OR DE "CHILDREN of teenage mothers" OR DE "CHILDREN of the rich" OR DE "CHILDREN of unemployed parents" OR DE "CHILDREN of unmarried parents" OR DE "CHILDREN of working parents" OR DE "CHRISTIAN children" OR DE "CHURCH group work with children" OR DE "CITY children" OR DE "COMIC books & children" OR DE "COMPUTERS & children" OR DE "DEAFBLIND children" OR DE "EXCEPTIONAL children" OR DE "FIRST-born children" OR DE "FOSTER children" OR DE "GIRLS" OR DE "GRANDCHILDREN" OR DE "HINDU children" OR DE "HOMELESS children" OR DE "IMMIGRANT children" OR DE "INDIGENOUS children" OR DE "INFANTS" OR DE "INTERNET & children" OR DE "JEWISH children" OR DE "MASS media & children" OR DE "MEDICALLY uninsured children" OR DE "MIDDLE children" OR DE "MISSING children" OR DE "MOTION pictures & children" OR DE "MUSIC & children" OR DE "MUSLIM children" OR DE "NEWSPAPERS & children" OR DE "ONLY child" OR DE "ORPHANS" OR DE "OVERWEIGHT children" OR DE "PASSIVE smoking in children" OR DE "PLAYMATES" OR DE "POLICE services for juveniles" OR DE "POOR children" OR DE "POSTHUMOUS children" OR DE "PRESCHOOL children" OR DE "PROBLEM children" OR DE "RACIALLY mixed children" OR DE "RADIO & children" OR DE "REFUGEE children" OR DE "RUNAWAY children" OR DE "RURAL children" OR DE "SCHOOL children" OR DE "SECOND-born children" OR DE "SELF-defense for children" OR DE "SOCIAL case work with children" OR DE "STEPCHILDREN" OR DE "STREET children" OR DE "SUBURBAN children" OR DE "TELEVISION & children" OR DE "TELEVISION advertising & children" OR DE "THIRD culture children" OR DE "TRAFFIC safety & children" OR DE "VAGRANT children" OR DE "VIDEO games & children" OR DE "WHITE children" OR DE "YOUNGEST child" OR DE "ADOLESCENCE" OR DE "TEENAGERS" OR DE "YOUNG adults" OR DE "CHILDREN" OR DE "MINORS" OR DE "YOUTH" \| 92,996 \| \| S11 \| child* or youth or youths or adolescen* or pubescen* or prepubescen* or "high school*" or "middle school* or teen* or tween or tweenie* or tweens or "young adult*" \| 437,871 \| \| S12 \| S10 OR S11 \| 441,538 \| \| S13 \| girl* or schoolgirl* or "young women" or "young woman" or marriageable or female* or feminin* or menstru* or pregnan* \| 175,273 \| \| S14 \| DE "GIRLS" OR DE "FATHER-daughter relationship" OR DE "FEMALE juvenile delinquents" OR DE "HOMELESS girls" OR DE "INFANT girls" OR DE "JEWISH girls" OR DE "MASS media & girls" OR DE "MINORITY girls" OR DE "MUSLIM girls" OR DE "POOR girls" OR DE "RURAL girls" OR DE "SCHOOLGIRLS" OR DE "SEXUALLY abused girls" OR DE "SISTERS" OR DE "TEENAGE girls" \| 3,632 \| \| S15 \| S13 OR S14 \| 175,439 \| \| S16 \| S3 AND S4 AND S9 AND S12 AND S15 \| 44 \| |
| --- | --- | --- | --- | --- | --- | --- | --- | --- | --- | --- | --- | --- | --- | --- | --- | --- | --- | --- | --- | --- | --- | --- | --- | --- | --- | --- | --- | --- | --- | --- | --- | --- | --- | --- | --- | --- | --- | --- | --- | --- | --- | --- | --- | --- | --- | --- | --- | --- | --- | --- | --- |
|  |

**SCOPUS Searched December 30, 2020 result=92**

((( ("underage marriage*" or "forced marriage*" or "child bride*" or "honor killing*" or "honour killing*" or (abduct* W/3 marriage*) or "forced sex*" or prostitut* or "bride burning" or "domestic violence" or "intimate partner violence" or IPV or dowry or fmg or "genital mutilation*" or sti or "sexually transmitted" or std or condom* or contracept* or "birth control*")) or ( ((sex) w/2 (empower* or control* or choice* or decision* or deciding or decide or autonomy or independence or rights or "self assurance" or liberation or "self actuali*" or "self sureness" or education or health)) or ((sexual* or reproductive) w/3 (empower* or control* or choice* or decision* or deciding or decide or autonomy or independence or rights or "self assurance" or liberation or "self actuali*" or "self sureness" or education or health))) or ( (digital* w/2 (empower* or literate or literacy)))) and ((girl* or schoolgirl*) or ( (child* or youth or youths or adolescen* or pubescen* or prepubescen* or "high school*" or "middle school*" or teen* or tween or tweenie* or tweens or "young adult*") and ("young women" or "young woman" or marriageable or female* or feminin*))) and ( ("cell phone*" or cellphone* or smartphone* or "smart phone*" or internet or webpage* or "web page*" or website* or "web site*" or mhealth or ehealth or telehealth or "tele health" or "m health" or "e health" or app or apps or tweets or tweeting or blog or blogging or blogger or texting or "text messag*" or "social media" or "online social network*" or "Baidu Tieba" or Douban or Facebook or Foursquare or "Google app*" or Influenster or Instagram or Kuaishou or Lasso or Linkedin or "Messenger" or Meetup or Mocospace or Snapchat or "snap chat" or Pinterest or Qzone or "Sina Weibo" or Skype or Steemit or "Tencent QQ" or "Tik Tok"or Tiktok or Tinder or Tumblr or Twitter or YouTube or "You Tube" or Reddit or Vero or Viber or SMS or VKontakte or Wattpad or Wechat or WhatsApp or Xanga or XING)) and ( ( "developing countr*" or "developing nation" or "developing nations" or Afghanistan or Albania or Algeria or Angola or Antigua or Barbuda or Argentina or Armenia or Aruba or Azerbaijan or Bahamas or Bahrain or Bangladesh or Barbados or Belarus or Belize or Benin or Bhutan or Bolivia or Bosnia or Herzegovina or Botswana or Brazil or Brunei or Bulgaria or "Burkina Faso" or Burundi or Cambodia or Cameroon or "Cape Verde" or "Central African Republic" or Chad or Chile or China or Colombia or Comoros or Democratic or Congo or "Costa Rica" or "Cote d'Ivoire" or Djibouti or Dominica or "Dominican Republic" or Ecuador or Egypt or "El Salvador" or "Equatorial Guinea" or Eritrea or Eswatini or Ethiopia or Fiji or Gabon or Gambia or (Georgia AND not (USA or University)) or Ghana or Grenada or Guatemala or Guinea or Guinea-Bissau or Guyana or Haiti or Honduras or Hungary or India or Indonesia or Iran or Iraq or Jamaica or Jordan or Kazakhstan or Kenya or Kiribati or Kosovo or Kuwait or Kyrgyzstan or Laos or Lebanon or Lesotho or Liberia or Libya or Macedonia or Madagascar or Malawi or Malaysia or Maldives or Mali or "Marshall Islands" or Mauritania or Mauritius or Mexico or Micronesia or Moldova or Mongolia or Montenegro or Morocco or Mozambique or Myanmar or Namibia or Nauru or Nepal or Nicaragua or Niger or Nigeria or Oman or Pakistan or Palau or Panama or Papua or "New Guinea" or Paraguay or Peru or Philippines or Poland or Qatar or Romania or Russia or Rwanda or "Saint Kitts" or Nevis or "Saint Lucia" or "Saint Vincent" or Grenadines or Samoa or "Sao Tome" or "Saudi Arabia*" or Senegal* or Serbia or Seychelles or "Sierra Leone" or "Solomon Islands" or Somalia or "South Africa" or "South Sudan" or "Sri Lanka" or Sudan or Suriname or Swaziland or Syria or Tajikistan or Tanzania or Thailand or Timor-Leste or Togo or Tonga or Trinidad or Tobago or Tunisia or Turkey or Turkmenistan or Tuvalu or Uganda or Ukraine or "United Arab Emirates" or Uruguay or Uzbekistan or Vanuatu or Venezuela or Vietnam or Yemen or Zambia or Zimbabwe ))) and not (( INDEX ( medline OR embase ) OR PMID ( 1* OR 2* OR 3* OR 4* OR 5* OR 6* OR 7* OR 8* OR 9* OR 0* ) )) [final "and not" string removes Medline and EMBASE records]

**PROQuest Dissertations and Theses Global Searched December 30, 2020 results =15**

((( ("underage marriage*" or "forced marriage*" or "child bride*" or "honor killing*" or "honour killing*" or (abduct* n/3 marriage*) or "forced sex*" or prostitut* or "bride burning" or "domestic violence" or "intimate partner violence" or IPV or dowry or fmg or "genital mutilation*" or sti or "sexually transmitted" or std or condom* or contracept* or "birth control*")) or ( ((sex) n/2 (empower* or control* or choice* or decision* or deciding or decide or autonomy or independence or rights or "self assurance" or liberation or "self actuali*" or "self sureness" or education or health)) or ((sexual* or reproductive) n/3 (empower* or control* or choice* or decision* or deciding or decide or autonomy or independence or rights or "self assurance" or liberation or "self actuali*" or "self sureness" or education or health))) or ( (digital* n/3 (empower* or literate or literacy)))) and ((girl* or schoolgirl*) or ( (child* or youth or youths or adolescen* or pubescen* or prepubescen* or "high school*" or "middle school*" or teen* or tween or tweenie* or tweens or "young adult*") and ("young women" or "young woman" or marriageable or female* or feminin*))) and ( ("cell phone*" or cellphone* or smartphone* or "smart phone*" or internet or webpage* or "web page*" or website* or "web site*" or mhealth or ehealth or telehealth or "tele health" or "m health" or "e health" or app or apps or tweets or tweeting or blog or blogging or blogger or texting or "text messag*" or "social media" or "online social network*" or "Baidu Tieba" or Douban or Facebook or Foursquare or "Google app*" or Influenster or Instagram or Kuaishou or Lasso or Linkedin or "Messenger" or Meetup or Mocospace or Snapchat or "snap chat" or Pinterest or Qzone or "Sina Weibo" or Skype or Steemit or "Tencent QQ" or "Tik Tok"or Tiktok or Tinder or Tumblr or Twitter or YouTube or "You Tube" or Reddit or Vero or Viber or SMS or VKontakte or Wattpad or Wechat or WhatsApp or Xanga or XING)) and ( ( "developing countr*" or "developing nation" or "developing nations" or Afghanistan or Albania or Algeria or Angola or Antigua or Barbuda or Argentina or Armenia or Aruba or Azerbaijan or Bahamas or Bahrain or Bangladesh or Barbados or Belarus or Belize or Benin or Bhutan or Bolivia or Bosnia or Herzegovina or Botswana or Brazil or Brunei or Bulgaria or "Burkina Faso" or Burundi or Cambodia or Cameroon or "Cape Verde" or "Central African Republic" or Chad or Chile or China or Colombia or Comoros or Democratic or Congo or "Costa Rica" or "Cote d'Ivoire" or Djibouti or Dominica or "Dominican Republic" or Ecuador or Egypt or "El Salvador" or "Equatorial Guinea" or Eritrea or Eswatini or Ethiopia or Fiji or Gabon or Gambia or (Georgia AND not (USA or University)) or Ghana or Grenada or Guatemala or Guinea or Guinea-Bissau or Guyana or Haiti or Honduras or Hungary or India or Indonesia or Iran or Iraq or Jamaica or Jordan or Kazakhstan or Kenya or Kiribati or Kosovo or Kuwait or Kyrgyzstan or Laos or Lebanon or Lesotho or Liberia or Libya or Macedonia or Madagascar or Malawi or Malaysia or Maldives or Mali or "Marshall Islands" or Mauritania or Mauritius or Mexico or Micronesia or Moldova or Mongolia or Montenegro or Morocco or Mozambique or Myanmar or Namibia or Nauru or Nepal or Nicaragua or Niger or Nigeria or Oman or Pakistan or Palau or Panama or Papua or "New Guinea" or Paraguay or Peru or Philippines or Poland or Qatar or Romania or Russia or Rwanda or "Saint Kitts" or Nevis or "Saint Lucia" or "Saint Vincent" or Grenadines or Samoa or "Sao Tome" or "Saudi Arabia*" or Senegal* or Serbia or Seychelles or "Sierra Leone" or "Solomon Islands" or Somalia or "South Africa" or "South Sudan" or "Sri Lanka" or Sudan or Suriname or Swaziland or Syria or Tajikistan or Tanzania or Thailand or Timor-Leste or Togo or Tonga or Trinidad or Tobago or Tunisia or Turkey or Turkmenistan or Tuvalu or Uganda or Ukraine or "United Arab Emirates" or Uruguay or Uzbekistan or Vanuatu or Venezuela or Vietnam or Yemen or Zambia or Zimbabwe )))

**PROSPERO Searched December 30, 2020**

Line Search for Hits

#1 smartphone* or cellphone* or "cell phone*" or "smart phone*" or

internet or website* or "web page*" or "online influencer*" or "social media"

or "mobile phone*" or mhealth or ehealth or e-health or m-health 10429

#2 tweets or tweeting or blog or blogging or blogger or texting or

"text messag*" or "social media" or "online social network*" or "Baidu Tieba"

or Douban or Facebook or Foursquare or "Google app*" or Influenster or

Instagram or Kuaishou or Lasso or Linkedin or Messenger or Meetup or

Mocospace or Snapchat or "snap chat" or Pinterest or Qzone or "Sina Weibo"

or Skype or Steemit or "Tencent QQ" or Tik Tok or Tiktok or Tinder or Tumblr or

Twitter or YouTube or "You Tube" or Reddit or Vero or Viber or SMS or VKontakte

or Wattpad or Wechat or WhatsApp or Xanga or XING 2891

#3 #1 or #2 11288

#4 girl* or schoolgirl* 722

#5 (female* or feminine or women or woman) and (young or youth or adolescen* or pubescen* or prepubescen*) 3060

#6 #4 OR #5 3575

#7 "developing countr*" or "developing nation" or "developing nations" or

Afghanistan or Albania or Algeria or Angola or Antigua or Barbuda or Argentina

or Armenia or Aruba or Azerbaijan or Bahamas or Bahrain or Bangladesh or Barbados 1990

#8 belarus or Belize or Benin or Bhutan or Bolivia or Bosnia or Herzegovina

Botswana or Brazil or Brunei or Bulgaria or Burkina Faso or Burundi or Cambodia

or Cameroon 10348

#9 belarus or Belize or Benin or Bhutan or Bolivia or Bosnia 295

#10 Herzegovina or Botswana or Brazil or Brunei or Bulgaria or

Burkina Faso or Burundi or Cambodia or Cameroon 10217

#11 "cape verde" 52

#12 "central african republic" 89

#13 Chad or Chile or China or Colombia or Comoros or Democratic or Congo 18487

#14 "costa rica" or "cote d'ivoire" or djibouti or domiinica or "dominical republic" 174

#15 Ecuador or Egypt 993

#16 "el salvador" or "eqatorial guinea" or eritrea 148

#17 Eswatini or Ethiopia or Fiji or Gabon or Gambia 1377

#18 Ghana or Grenada or Guatemala or Guinea or Guinea-Bissau 415

#19 Guyana or Haiti or Honduras or Hungary or India or Indonesia or Iran

or Iraq or Jamaica or Jordan or Kazakhstan or Kenya or Kiribati or Kosovo

or Kuwait or Kyrgyzstan or Laos or Lebanon or Lesotho or Liberia or Libya

or Macedonia or Madagascar or Malawi or Malaysia or Maldives or Mali or

"Marshall Islands" or Mauritania or Mauritius or Mexico or Micronesia or

Moldova or Mongolia or Montenegro or Morocco or Mozambique or Myanmar

Namibia or Nauru or Nepal or Nicaragua or Niger or Nigeria or Oman or

or Palau or Panama or Papua 9091

#20 "new guinea" 59

#21 Paraguay or Peru or Philippines or Poland or Qatar or Romania or

Russia or Rwanda or "Saint Kitts" or Nevis or "Saint Lucia" or "Saint Vincent"

or Grenadines or Samoa 1792

#22 "Saudi Arabia*" 629

#23 Senegal* or Serbia or Seychelles or "Sierra Leone" 228

#24 "Solomon Islands" or Somalia or "South Africa" 1217

#25 "South Sudan" or "Sri Lanka" 320

#26 Sudan or Suriname or Swaziland or Syria or Tajikistan or Tanzania or Thailand 1177

#27 Timor-Leste or Togo or Tonga or Trinidad or Tobago or Tunisia or Turkey or Turkmenistan or Tuvalu or Uganda or Ukraine or United Arab Emirates or Uruguay or Uzbekistan or Vanuatu or Venezuela or Vietnam or Yemen or Zambia or Zimbabwe 1613

#28 #7 or #8 or #9 or #10 or #11 or #12 or #13 or #14 or #15 or #16 or #17 or #18

or #19 or #20 or #21 or #22 or #23 or #24 or #25 or #26 or #27 42614

#40 (reproductive or sexual* or sti or std or "sexually transmitted" or

abortion* or "reproductive health" or rape or "forced marriage*" or "child bride*"

or "underage marriage*" or contracept* or "birth control" or condom* or pregnan* or menstrua*):TI 3073

#43 #13 AND #35 AND #37 AND #40 23
